# The conservation and functionality of the oxygen-sensing enzyme Factor Inhibiting HIF (FIH) in non-vertebrates

**DOI:** 10.1371/journal.pone.0216134

**Published:** 2019-04-29

**Authors:** Rachel J. Hampton-Smith, Briony A. Davenport, Yagnesh Nagarajan, Daniel J. Peet

**Affiliations:** School of Biological Sciences, University of Adelaide, Adelaide, South Australia, Australia; University of Liverpool, UNITED KINGDOM

## Abstract

The asparaginyl hydroxylase, Factor Inhibiting HIF (FIH), is a cellular dioxygenase. Originally identified as oxygen sensor in the cellular response to hypoxia, where FIH acts as a repressor of the hypoxia inducible transcription factor alpha (HIF-α) proteins through asparaginyl hydroxylation, FIH also hydroxylates many proteins that contain ankyrin repeat domains (ARDs). Given FIH’s promiscuity and the unclear functional effects of ARD hydroxylation, the biological relevance of HIF-α and ARD hydroxylation remains uncertain. Here, we have employed evolutionary and enzymatic analyses of FIH, and both HIF-α and ARD-containing substrates, in a broad range of metazoa to better understand their conservation and functional importance. Utilising *Tribolium castaneum* and *Acropora millepora*, we provide evidence that FIH from both species are able to hydroxylate HIF-α proteins, supporting conservation of this function beyond vertebrates. We further demonstrate that *T*. *castaneum* and *A*. *millepora* FIH homologs can also hydroxylate specific ARD proteins. Significantly, FIH is also conserved in several species with inefficiently-targeted or absent HIF, supporting the hypothesis of important HIF-independent functions for FIH. Overall, these data show that while oxygen-dependent HIF-α hydroxylation by FIH is highly conserved in many species, HIF-independent roles for FIH have evolved in others.

## Introduction

In mammals, communication of oxygen availability within cells is achieved in part by enzymes which directly use dioxygen as a co-substrate [1]. One such enzyme is the oxygen and 2-oxoglutarate (2-OG)-dependent dioxygenase, Factor Inhibiting HIF (FIH), an asparaginyl hydroxylase first characterised through its role in modulation of the Hypoxia-Inducible Factor (HIF) transcription factors [2–5].

**Fig 1:**
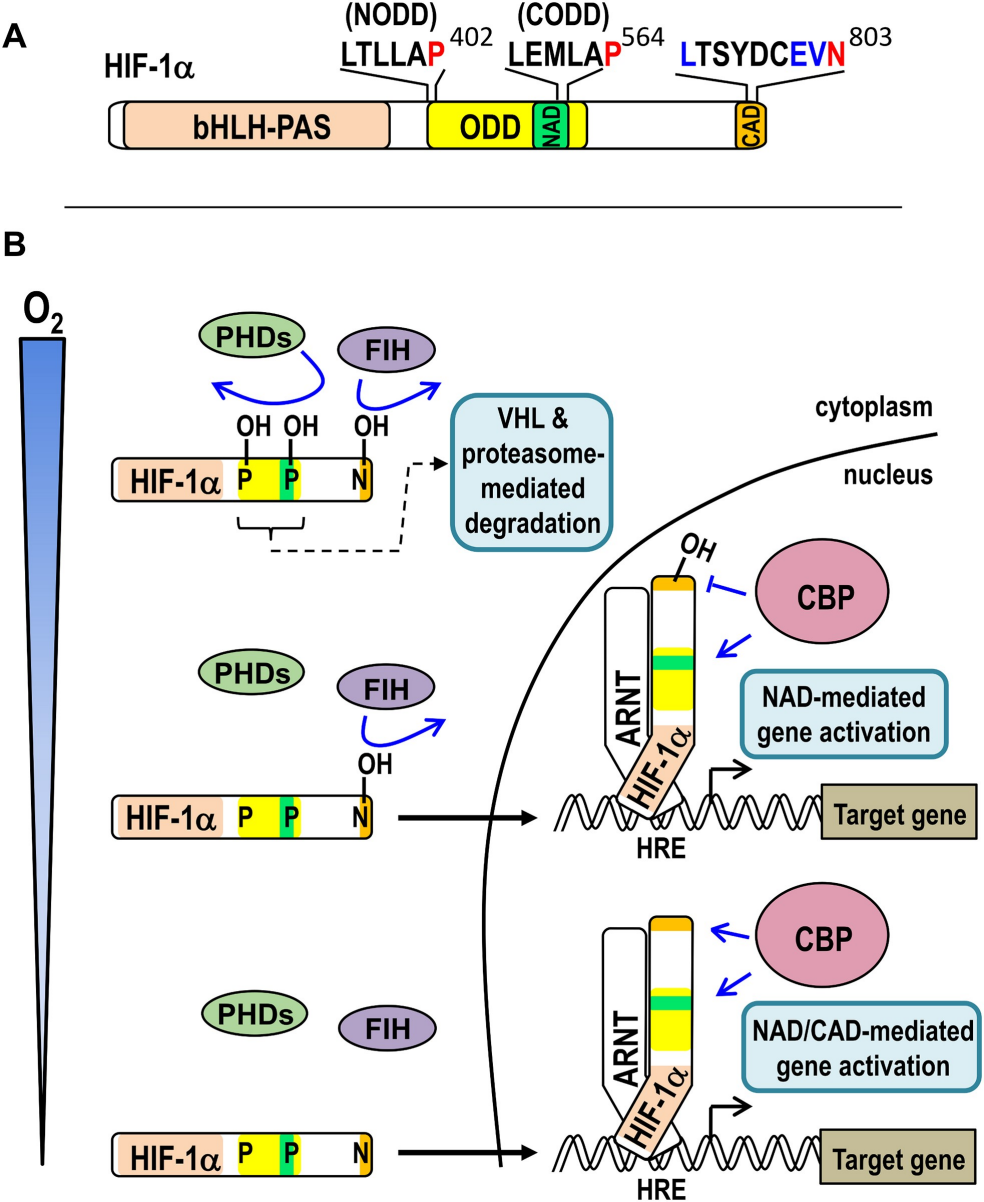
Activation of HIF-1 signalling by hypoxia. (A) Schematic of human HIF-1α (hsHIF-1α) showing the regions involved in DNA-binding and dimerisation with ARNT (bHLH-PAS), oxygen-dependent degradation (ODD), and coactivator binding (the N- and C-terminal transactivation domains, NAD and CAD, respectively). The asparaginyl residue (Asn803) hydroxylated by FIH is shown in red with residues constituting the remainder of the “FIH preferred target sequence” shown in blue above the CAD. The PHD-targeted prolyl residues which are central to the N- and C-terminal ODDs (Pro402 (NODD) and Pro564 (CODD), respectively) are similarly indicated above the ODD. (B) Schematic showing the consequences of different oxygen levels (from “adequate” or normoxic at the top of the schematic to severely hypoxic at the bottom) on FIH/PHD enzyme and hsHIF-1α activity. When adequate oxygen is present, the PHDs and FIH are both active, resulting in hydroxylation of their target residues in HIF-1α (coloured as in part A). Prolyl hydroxylation results in efficient VHL-mediated ubiquitination and rapid proteasomal degradation of HIF-1α, thus ensuring minimal HIF-1 target gene activation. At intermediate levels of oxygen, the PHDs are inactive, resulting in HIF-1α stabilisation, translocation to the nucleus, and partnering with ARNT on hypoxia response elements (HREs). Ongoing FIH-mediated hydroxylation at this oxygen tension, however, precludes CBP binding to the CAD, thus only the NAD recruits CBP for target gene activation. Under more severe hypoxia, both PHDs and FIH are inactive, thus both the NAD and CAD of HRE-bound HIF-1α can recruit CBP for target gene activation.

The HIFs, which are master regulators of the genomic response to hypoxia (oxygen insufficiency), are dimers of two basic helix-loop-helix-Per ARNT Sim homology (bHLH-PAS) transcription factors: an oxygen-responsive α-subunit (Fig 1A), which may be any of HIF-1α, -2α or -3α, and a common, constitutively active β-subunit, also known as the aryl hydrocarbon nuclear translocator (ARNT, reviewed in [6]). Under conditions of adequate oxygenation (normoxia), HIF-1α and HIF-2α both interact with FIH via their C-terminal transactivation domain (CAD), wherein FIH hydroxylates a specific asparagine (Asn) residue (Asn803 in human HIF-1α (hsHIF-1α)), thereby repressing transcriptional activity by preventing p300/CBP coactivator recruitment (Fig 1B) [2–4]. In addition, three oxygen-sensing prolyl hydroxylase enzymes from the same enzyme family of dioxygenases, PHDs 1–3, target two oxygen-dependent degradation domains (the NODD and CODD) within the HIF-α proteins (Fig 1B). PHD-mediated prolyl hydroxylation of the HIF-α proteins facilitates Von Hippel Lindau protein (VHL)-mediated ubiquitination and rapid proteasomal degradation [7–9]. Together, the activity of FIH and the PHDs ensures tight repression of HIF transcriptional activity in normoxia. When oxygen levels are limiting however, the activity of the oxygen-dependent HIF hydroxylases is reduced, and the stable unhydroxylated HIF-α protein translocates to the nucleus. There, it partners with ARNT, binds to hypoxic response elements in the regulatory regions of target genes, and efficiently recruits p300/CBP coactivators to initiate transcription (Fig 1B). Thus, in humans, both the PHDs and FIH are thought to act as primary cellular oxygen sensors that mediate cellular responses to hypoxia.

More recently, FIH’s substrate repertoire has been extended to include numerous proteins harbouring an ankyrin repeat domain (ARD), including IκBα, Notch1-3, Asb4, Tankyrase-2, Ankyrin R, TRPV3 and RIPK4 [10–17]. ARDs contain multiple ankyrin repeats, a distinct structural motif, and commonly mediate protein-protein interacations. Importantly, FIH’s preferred target sequence, LXXXXX[D/E]VN [11], is common to many ankyrin repeats, thus implying that this group of substrates is extensive [18]. While a small number of confirmed ARD targets have shown functional changes in response to altering FIH activity (e.g. [14–16]), the majority appear to be unaffected by hydroxylation. As a result, the biological importance of widespread FIH/ARD interactions is currently unclear. One possibility is that FIH’s substrate promiscuity is a trade-off for achieving a small number of functionally important, but as yet undefined, ARD interactions. Alternatively, it has been proposed that ARDs function collectively as a “sink” which sequesters FIH protein away from HIF-1α, thus regulating HIF activity [12, 18]. Like all enzymes, FIH favours binding to unhydroxylated substrate over hydroxylated product [17, 19], thus both the number of ARD proteins and the level of hydroxylation of the ARDs within a cell is hypothesised to determine the effectiveness of the sink. In this way, fluctuating oxygen levels may regulate not just FIH catalytic activity, but also its availability through variable sequestration, both of which can influence HIF-α activity [20, 21].

Due to the sheer volume of predicted ARD substrates for FIH [11], determining the functional significance of individual as well as global ARD hydroxylation has proved a challenge. In addition, the influence of FIH on the HIF pathway appears to be gene-specific [20, 22], an observation that remains poorly understood. Thus, the relative contribution of both HIF-α and ARD substrate classes to FIH’s physiological function as a metabolic regulator [22] remains uncertain. To gain new insight, analysis of the evolutionary conservation of FIH-HIF and FIH-ARD interplay can provide invaluable information on the importance of these interactions. Current literature suggests that FIH homologs are limited to a subset of Metazoan species [23]. This pattern of conservation differs from that of ARDs, which are known to be present in many kingdoms of life [24]. It also differs from HIF-α, which is present in numerous, if not all, Metazoa (see for example [25–32]). Notably however, it is in strong agreement with the existence of CAD-containing homologs of HIF-α. In accordance with this observation, a tight functional link is assumed to exist between FIH and the HIF-α CAD [23]. Nonetheless, detailed molecular analysis of HIF-α regulation by HIF hydroxylases is limited to only a few invertebrate species, including *Drosophila melanogaster* (fruit fly) [7, 33–41], *Caenorhabditis elegans* (nematode) [8, 42], and *Trichoplax adhaerens* (placozoan) [23]. In each of these systems, a HIF/PHD/VHL axis is conserved. Indeed, the work examining the best characterised of these systems, that in *C*. *elegans*, was instrumental in identifying the first oxygen-regulated PHD and its homologs in mammals [8]. It is important to note, however, that each of these invertebrates lack both FIH and a HIF-α CAD. Thus, hydroxylation of the HIF-α CAD by FIH from an invertebrate species is yet to be demonstrated. Indeed, if such a modification is absent when ARD hydroxylation is conserved, this would represent strong evidence of a significant, more conserved, and possibly more important role for ARD-FIH interactions.

**Fig 2:**
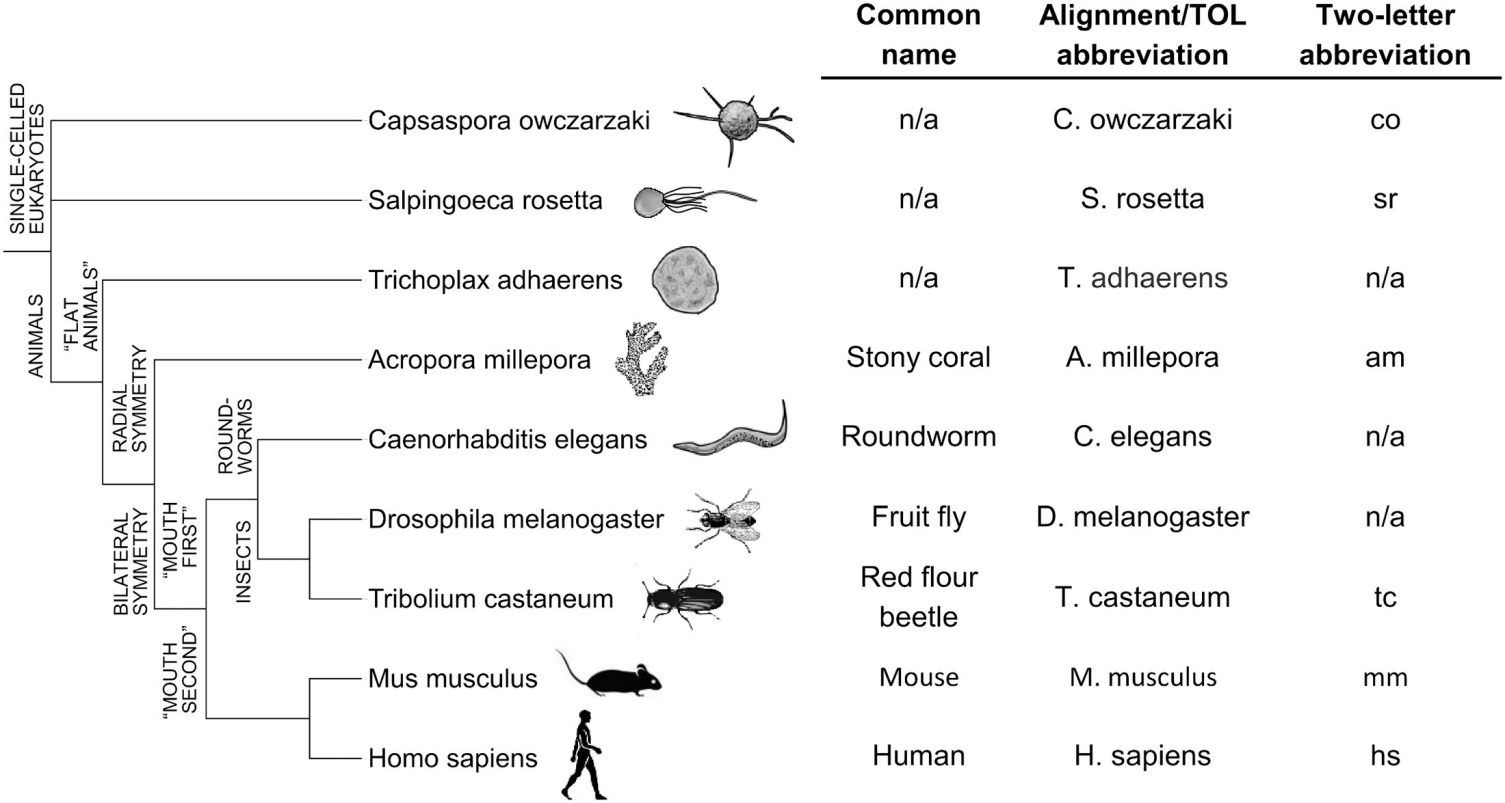
Evolutionary relationships and nomenclature of key eukaryotic species. Species which have previously undergone detailed molecular analysis of their HIF pathway components, including Caenorhabditis elegans [8], Drosophila melanogaster [34] and Trichoplax adhaerens [23], are shown in a cladogram with biochemically characterised species in the current work. An adjacent table shows species name abbreviations used in Fig 3 and sequence alignment figures, as well as two-letter abbreviations utilised for concise reference to various protein homologs (e.g. Tribolium castaneum FIH is abbreviated to “tcFIH”). A more comprehensive list of species name abbreviations can be found in S1 Table. n/a = not applicable.

In this report we utilised currently available sequencing data to conduct an in-depth examination of FIH and HIF-α CAD conservation among the Eukaryota. While co-conservation of both FIH and HIF-α CAD within Metazoan species was common, evidence was also found supporting a measure of independence of the two sequences during evolution. In addition, we sought to define the HIF-α CAD and ARD hydroxylation characteristics of a several invertebrate FIH homologs. The evolutionary relationships of these biochemically examined species and their naming conventions in this manuscript are detailed in Fig 2. Through functional testing of these enzymes, we provide the first evidence of active FIH enzymes outside of the Vertebrata, and demonstrate that ARD hydroxylation is likely conserved among metazoan FIHs, while HIF-α CAD modulation is less consistently conserved. Collectively, these data demonstrate that FIH is an enzyme with ancient origins, whose primary function likely varies across evolutionary history.

## Results

### Identification of eukaryotic HIF-α CAD and FIH homologs

In order to investigate the importance of HIF-α CAD-FIH interactions, we first examined FIH and HIF-α CAD conservation patterns in Eukaryota. Only species which had undergone large-scale sequencing of their genome or transcriptome were included in the analysis, unless both FIH and HIF-α CAD homologs could be found amongst less comprehensive sequence submissions. To locate HIF-α or FIH homologs, sequence databases were searched using the BLASTp and tBLASTn algorithms [43] and either full length human FIH (hsFIH) or a variety of HIF-α homologs (either full length or encompassing only the C-terminal 46 amino acids). As the N-terminal bHLH-PAS domain of HIF-α is the only region to consistently be detected by BLAST in cross-species searches, downstream sequence from bHLH-PAS BLAST hits was often further analysed for the NODD, CODD and CAD using HMMER [44] and hidden Markov models (HMMs) generated from identified HIF-α homologs. A sequence was only classified as HIF-α if it contained a full or partial (if 5’ truncated) match to the bHLH-PAS, as well as at least one of the NODD, CODD or CAD in the same sequence or numerically neighbouring genomic contigs. The only exception to this rule was the putative HIF-α homolog from the sponge, *Amphimedon queenslandica*, which showed similarity to hsHIF-1α only in the bHLH-PAS region.

#### Analysis of HIF-α and CAD conservation

**Fig 3:**
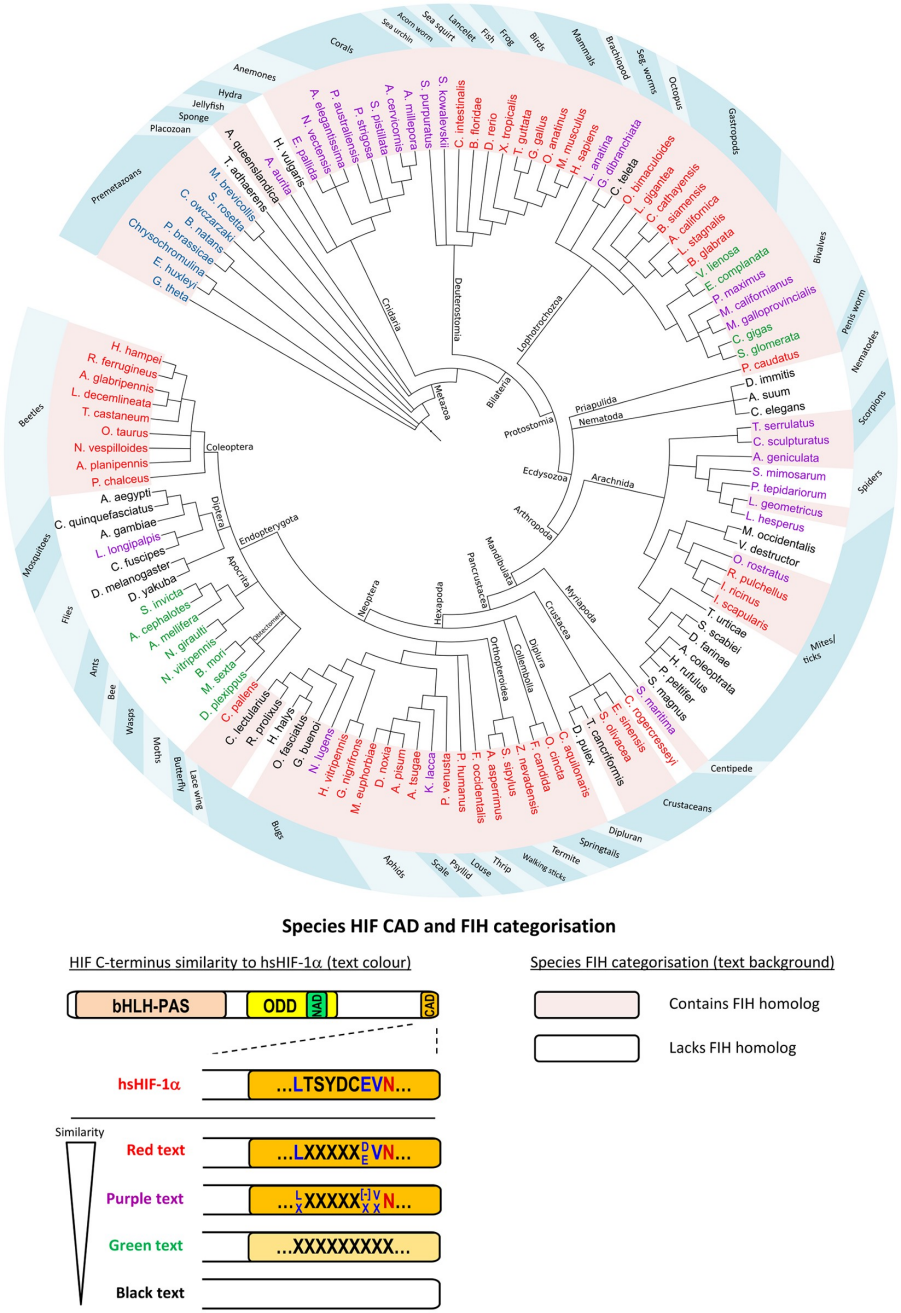
Conservation of HIF-α CAD and FIH across the Eukaryota. BLAST and hidden markov models were used to identify HIF-α and FIH homologs in a wide variety of Eukaryotes. Evolutionary relationships between representative analysed species are indicated by a cladogram. An explanation of species name background shading and text colour is provided in a schematic below the cladogram. Background shading of species names is used to indicate FIH status, with pink and white shading representing FIH-containing and FIH-lacking species, respectively. Amongst Metazoa, the colour of the text for the name of each species (red, purple, green or black) refers to its HIF-α characteristics, specifically, its level of C-terminal similarity to that of hsHIF-1α CAD. The features which define the four C-terminus types are represented pictorially using an orange-shaded CAD and text within the CAD to indicate the high similarity of CBP or FIH-binding residues, respectively. For comparison, the hsHIF-1α CAD is shown in the schematic coloured orange, with its FIH target sequence shown within the CAD using the same colouring as Fig 1. Red coloured species in the cladogram have strong CBP-binding residue conservation, and also contain hsFIH’s preferred target sequence. Purple species also have strong CBP-binding residue conservation, but at least 1 or more of the residues in FIH’s preferred target sequence (excluding the Asn) are not conserved (indicated by “X”s), which may render the protein an inefficient FIH substrate [47, 48]. “[–]”indicates an acidic residue (Asp or Glu). Green species have only moderate CBP-binding residue conservation (indicated by pale orange colouring of the CAD) and no FIH target sequence, while black species have no recognisable CAD. Premetazoans with blue text in the cladogram lack a HIF-α homolog. Cladogram branches are labelled with taxonomic classifications. The blue ring outside the cladogram indicates species common names. Cladogram tree generated using phyloT and displayed using Interactive Tree of Life [49].

HIF-α BLAST/HMMER searches were performed on hundreds of species from single-celled eukaryotes to mammals. Of these, nearly 200 produced matches to at least one domain among the bHLH-PAS, NODD, CODD or CAD, with 119 species meeting our organism inclusion/HIF-α classification requirements. In agreement with previous reports (see e.g. [23, 25]), analysis of the identified HIF-α homologs suggested that this transcription factor is restricted to metazoan Eukaryotes (Fig 3). To identify CAD conservation patterns within these homologs, attention was subsequently focussed on the HIF-α homolog C-termini. The CAD plays a role in vertebrate HIF target gene activation by binding the CBP/p300 family of coactivator proteins. This interaction is oxygen-sensitive due to FIH-mediated asparaginyl hydroxylation of the CAD [4]. Thus, for newly-identified HIF-α homologs, it was of interest to determine if their C-termini retained (1) CBP binding residues [45, 46], and (2) a likely FIH target motif [11]. Analysis of these characteristics led to classification of four different C-terminus types (represented by red, purple, green and black species names in Fig 3), which are delineated by their apparent level of divergence from the hsHIF-1α CAD. The criteria used to define each classification are described in more detail below.

At the “most divergent” end of the scale were HIF-α homologs which had no detectable CAD-like sequence (represented with black species names in Fig 3). In agreement with published evolutionary analyses, this group included *D*. *melanogaster*, *Anopheles gambiae* (mosquito), *C*. *elegans*, *Daphnia pulex* (water flea) and *T*. *adhaerens* [23, 25]. It was additionally found that various subgroups of flies, mites, ticks, “bugs”, and more simple species such as *A*. *queenslandica* and *Hydra vulgaris* (hydra) also lacked a CAD (hereafter referred to as CAD^—^species), suggesting that their HIF-α homologs lack C-terminal transactivation capacity, or achieve transactivation independent of CBP.

**Fig 4:**
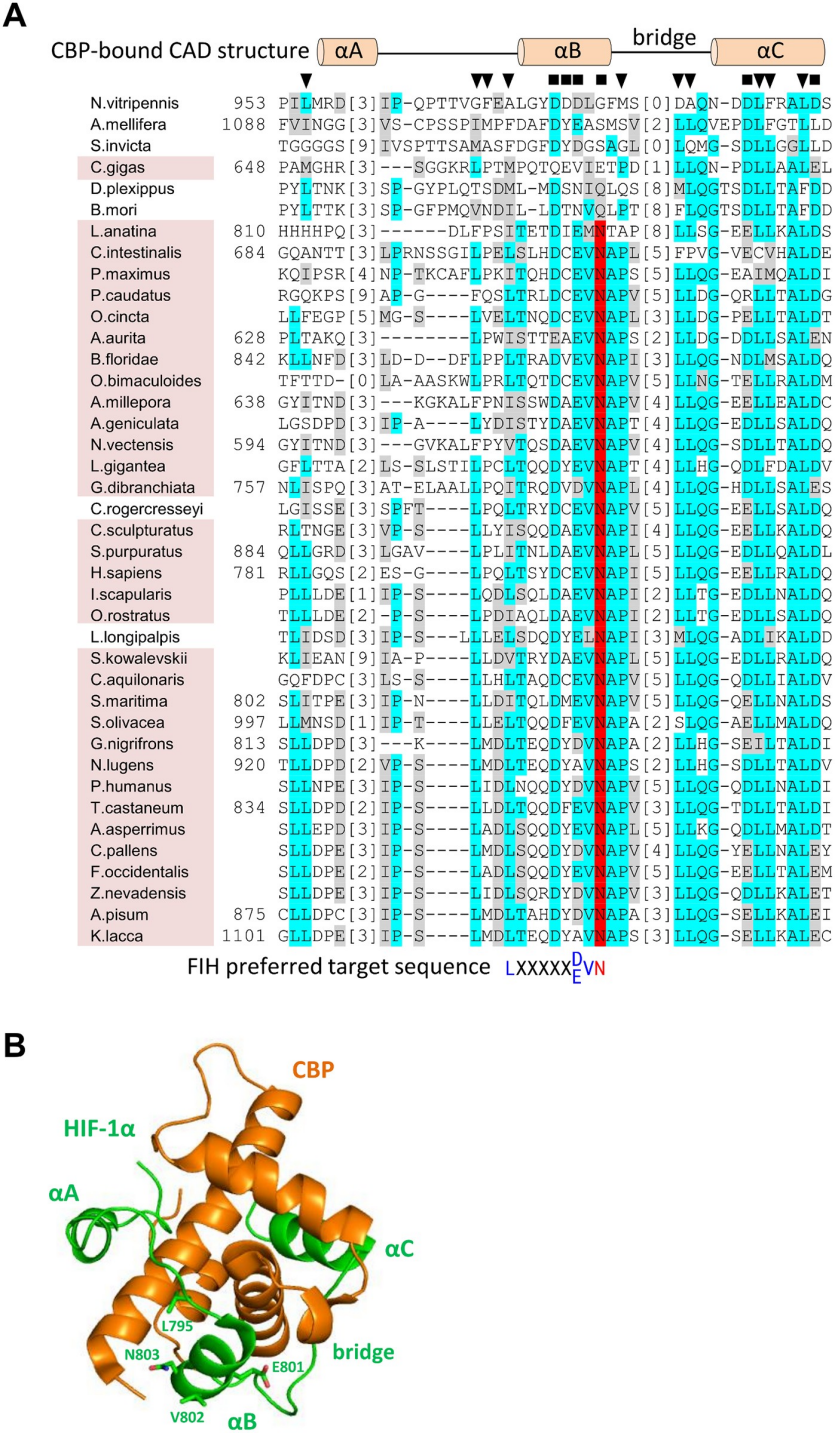
Conservation of CBP and FIH binding residues in HIF-α CAD homologs. (A) Known and putative HIF-α CAD sequences from representative species were aligned using MUSCLE [50]. Identical and similar residues are indicated by cyan and grey highlights, respectively, while likely FIH target Asn residues are highlighted in red. To facilitate viewing of as large a CAD region as possible, low similarity regions of the alignment were deleted and replaced with bracketed numbers which indicate how many residues in each sequence are not shown. Amino acid numbers within the HIF-α proteins (where available) are shown to the left of the sequences. To compare functional regions of hsHIF-1α CAD with the aligned homologs, CBP and FIH-interacting residues are also depicted. Specifically, the secondary structure of hsHIF-1α CAD when bound to CBP [45] as well as residues predicted to be involved in polar (black squares) or hydrophobic (black triangles) interactions with CBP are shown above the alignment. The FIH preferred target sequence is shown below the alignment, coloured as for Fig 1. Conservation of FIH in a species is indicated by pink background shading of the species’ name, and correlates strongly with the level of sequence similarity to hsHIF-1α CAD. (B) The secondary structure depicted above the alignment in part A is shown in the context of the NMR structure of CBP (orange) bound to hsHIF-1α CAD (green) [45]. The different regions of hsHIF-1α CAD that interact with CBP, including helices αA-αC and the αB-αC bridge, and the FIH target sequence residue sidechains, Leu795, Glu801, Val802 and Asn803 are labelled with green text. Structure image generated using Pymol [Schrodinger, 2015 #1226] and PBD structure 1L8C [45].

In contrast to the CAD^—^species, BLAST/HMMER searches also revealed a wide variety of species with robust homology to the CAD of hsHIF-1α (hereafter referred to as CAD^+^ species). To determine if the HIF-CADs preserved CBP-binding residues, representative CAD sequences were first aligned using MUSCLE [50] (Fig 4A). Next, conserved features from the alignment were compared with key interaction residues in the HIF-1α/CBP NMR structure [45]. This structure demonstrates that the CAD forms three α-helices (“αA”, “αB” and “αC”) connected by extended sequences when it is bound to CBP (Fig 4B, and schematically represented above the alignment in Fig 4A) [45]. Comparing the CAD alignment from multiple species to these structural motifs revealed that residues corresponding to helix “αC” and the C-terminal end of the αB-αC “bridge” were the most consistently conserved, while those in the region around helix αB were less conserved (Fig 4A). Residues equivalent to the helix αA region showed little similarity. Importantly, in most cases the highly conserved residues corresponded well with those predicted to form important polar or hydrophobic contacts with CBP (indicated in Fig 4A above the alignment), suggesting that interaction of the CAD with CBP is conserved from vertebrates through to simple cnidarians.

The CAD^+^ species were next analysed for conservation of a FIH target sequence. In hsHIF-1α CAD, the FIH target motif resides within and just N-terminal to helix αB. In agreement with the observed conservation of CBP-binding residues in this region, many species contained a perfect match to the hsFIH preferred target sequence, LXXXXX[D/E]VN (indicated with red species names in Fig 3) [11]. Among those that were only a partial match to the to the preferred target sequence (i.e. lack at least one of the conserved residues, but retain the Asn, indicated by purple species names in Fig 3), the vast majority diverged at only a single residue, most commonly the -8 Leu (Fig 4A). Although the influence of the -8 Leu on hydroxylation has not been directly tested for HIF-α substrates, its conservation in both HIF-α and ARDs suggests it may be important for FIH binding. Hence, HIF-α CADs from purple species are predicted to bind CBP, but have uncertain efficiency as FIH substrates.

During the course of HIF-α homolog analysis, a combination of HIF-α sequence alignments and manual inspection led to the identification of an additional group of CAD-like sequences which were not detected by BLAST/HMMER searches (green species in Fig 3). These sequences, including those from the Apocrita (wasps, ants and bees), Obtectomera (moths and butterflies) and some bivalves (e.g. some mussels and oysters) showed considerable homology to hsHIF-1α CAD in the helix αC region, but were noticeably more disparate around helix αB. Because of this, these species lack a clear FIH target motif. Consequently, it is possible that HIF-α homologs from these species retain CBP binding, but are unlikely to be hydroxylated by FIH.

Overall, the predicted functional homology of the different CAD groups compared to the hsHIF-1α CAD is summarised in Table 1. It appears that both FIH and CBP-binding capacity across homologs may vary considerably, and not necessarily to the same degree in a single species (see for example *C*. *gigas* in Fig 4, which has moderate CBP binding residue conservation, but is very unlikely to be hydroxylated by FIH). The evolutionary pressures which lead to gain or loss of the CAD, as well as its “targetability” by FIH, are currently unknown. Examination of the evolutionary tree in Fig 3 suggests that the earliest appearance of CAD-like sequences within HIF-α occurs in a subset of Cnidaria. Subsequent to this, the CAD has been lost on numerous occasions throughout evolutionary history, resulting in an evolutionary tree with a fascinating patchwork of CAD retention (Fig 3). Obviously, one factor which may promote CAD conservation is the presence of FIH, hence the co-conservation of FIH was examined.

**Table 1:** Predicted CBP and FIH-binding capacity of HIF-α CAD homologs.

CAD classification*	Predicted CBP-binding capacity	Predicted efficiency as FIH substrate
Red	+++	+++
Purple	+++	++
Green	++	-
Black	-	-

Predictions are based on conservation of CBP-binding residues and the FIH preferred target sequence relative to hsHIF-1α CAD.

*Colours defined as for CAD categorisation in Fig 3.

+++ efficient binding/hydroxylation

++ moderate binding/hydroxylation

- no binding/hydroxylation

#### Conservation of FIH and comparison with HIF-α CAD retention

**Fig 5:**
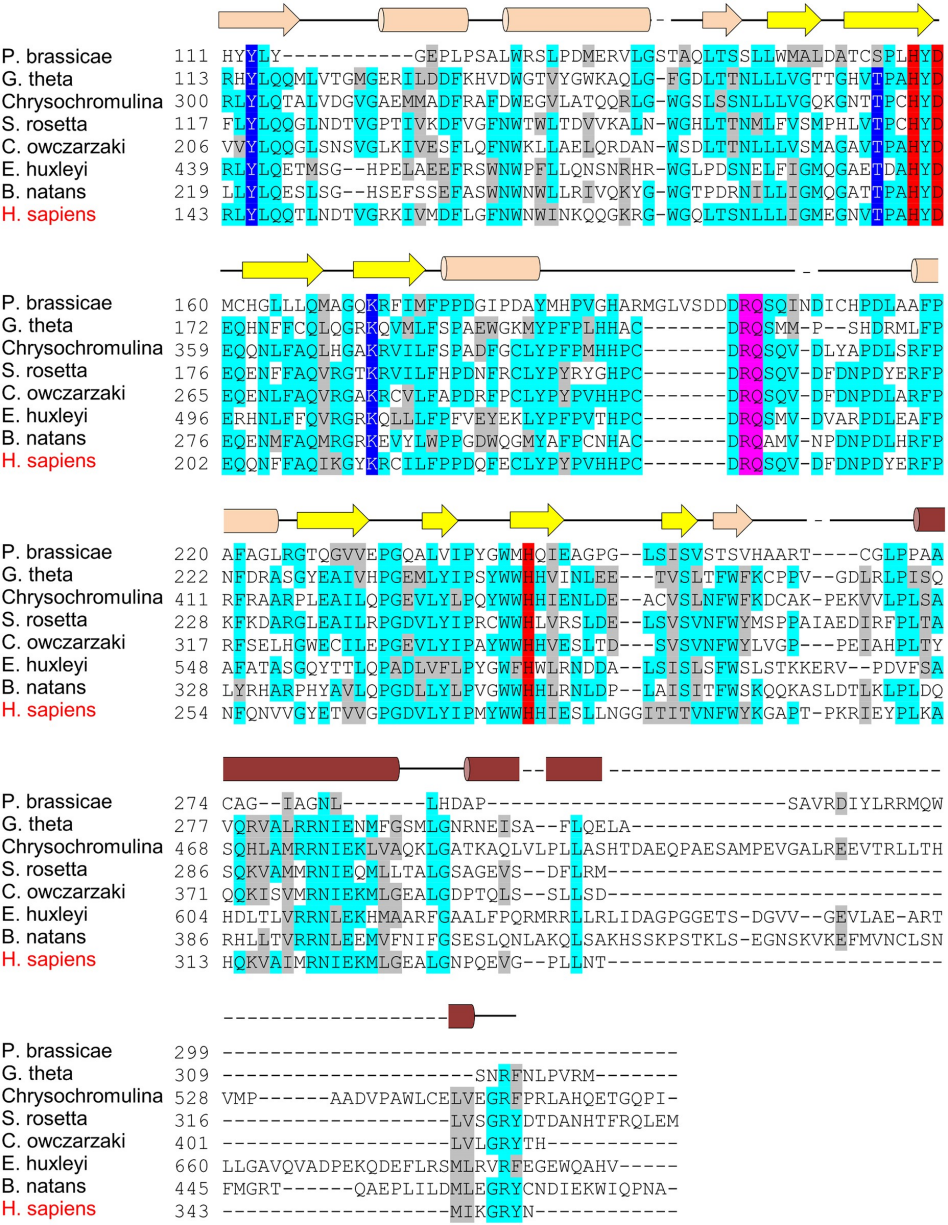
Alignment of putative premetazoan FIH homologs with human FIH. The sequences of FIH homologs C-terminal to the first 2-OG-binding residue (Tyr145 in hsFIH) were aligned using Clustal Omega [51]. Residues strongly or partially conserved are shown with cyan and grey highlights, respectively. Residues involved in iron coordination (red), 2-OG binding (dark blue), and target asparagine positioning (pink) are indicated. The secondary structure of hsFIH is depicted above the alignment, with yellow arrows indicating the beta strands which make up the double-stranded β-helix (DSBH), and dark brown helices denoting those involved in dimerisation. Amino acid numbers are shown to the left of the alignment. Alignment shading performed using the BoxShade Server. Species name abbreviations and sequence IDs can be found in S1 Table.

To locate novel FIH homologs, BLAST searches of the Eukaryota using the hsFIH sequence were employed. In order to be classified as FIH, candidates with an alignment score greater than 200 bits were required to conserve hsFIH iron- and target Asn-binding residues His199, Asp201, Arg238, Gln239 and His279, and the three 2-OG-binding residues Tyr145, Thr196 and Lys214. Analysis of the BLAST results revealed putative FIH homologs in a large variety of metazoan species (indicated by pink background shading of species’ names in Fig 3). Strikingly, in contrast to the distribution of HIF-α homologs, FIH hits were also located in a small number of premetazoan eukaryotes. To more closely scrutinise FIH structural conservation in the metazoan and premetazoan candidates, FIH protein sequences were collected or constructed and aligned by Clustal O [51]. Examination of the metazoan candidates demonstrated that residues involved in double-stranded β-helix (DSBH) formation and enzyme dimerisation were well-conserved (S1 File). Indeed, humans and the simple Porifera member, *A*. *queenslandica*, contain enzymes with 51% residue identity. Amongst the premetazoan candidates, those most closely related to animals, including sequences from the Opisthokonta *Salpingoeca rosetta* (Choanoflagellida) and *Capsaspora owczarzaki* (Ichthyosporea), also preserved FIH’s key structural features (Fig 5). For the more distantly related premetazoan sequences, including those from *Chrysochromulina* (Haptophyceae), *Bigelowiella natans* (Rhizaria) and *Guillardia theta* (Cryptophyta), structural conservation was more variable. For example, the predicted FIH homolog from *G*. *theta* was well conserved, while those from *B*. *natans* and *Chrysochromulina* contained a large insertion in the region involved in dimerisation (Fig 5). Given the critical role of hsFIH’s C-terminal dimerisation helices in catalysis [52], the functional homology of these more distantly related hits to hsFIH is less certain.

After classifying each species as FIH-containing or FIH–lacking (FIH^+^ or FIH^—^, respectively), the FIH conservation pattern across species was compared with that of the HIF-α CAD. Thus far, evolutionary studies have suggested that FIH and a CAD-containing HIF-α homolog are always co-conserved [23, 25]. However, our identification FIH homologs in premetazoans which lack HIF-α is contrary to this observation, and has significant implications. Indeed, if these sequences represent genuine enzyme homologs, it can be assumed that the original role of FIH was not hydroxylation of HIF-α. Moreover, disparate conservation of FIH and CAD sequences was perpetuated to a small degree in higher organisms. For example, it was observed that multiple species, including *A*. *queenslandica*, *Capitella teleta* (segmented worm), *Triops cancriformis* (tadpole shrimp) and *Cimex lectularius* (bed bug), were CAD^—^FIH^+^ (black species with pink background shading in Fig 3), immediately suggesting that FIH has a purpose beyond CAD regulation in these organisms. Similarly, a number of FIH^+^ bivalve molluscs were found to contain a CAD that lacked the FIH target motif (Fig 4A, see e.g. *Crassostrea gigas*), again pointing to alternative roles for FIH in these species. In contrast, many species showed a conservation pattern which, collectively, is supportive of a strong functional connection between FIH and the CAD. For example, absence of FIH in a number of species was accompanied by absence of the CAD domain in HIF-α (e.g. *Caenorhabditis elegans* (nematode), *Anopheles gambiae* (African malaria mosquito), and *Tetranychus urticae* (two-spotted spider mite)), or absence of a target Asn in the CAD-like sequence (e.g. the Apocrita (wasps, ants and bees)). Conversely, nearly all species with a CAD containing the FIH target sequence were found to also contain FIH (note the correlation between red/purple species and pink background shading in Fig 3), the only exceptions being the crustacean, *Caligus rogercresseyi* (sea louse), *Lutzomyia longipalpis* (sand fly), and a number of spiders. Overall, analysis of predicted FIH and HIF-α homologs suggests that the presence of FIH is frequently associated with conservation of a HIF-α CAD domain containing a target Asn. If this co-conservation equates to FIH-mediated regulation of the CAD, then such modulation is clearly an important characteristic of many Metazoan HIF-α homologs. Nonetheless, it is clear that FIH additionally functions beyond CAD regulation.

### The HIF-α homolog from the beetle, *Tribolium castaneum*, is regulated by FIH and PHD-mediated hydroxylation

To assess the functionality of the FIH/HIF-α CAD interaction in an invertebrate species, the *Tribolium castaneum* (red flour beetle) was examined. This species was chosen due to the strong conservation of the hsFIH preferred target sequence in the HIF-α CAD (Fig 4A), which provided an opportunity to determine if sequence conservation equated to functional conservation.

**Fig 6:**
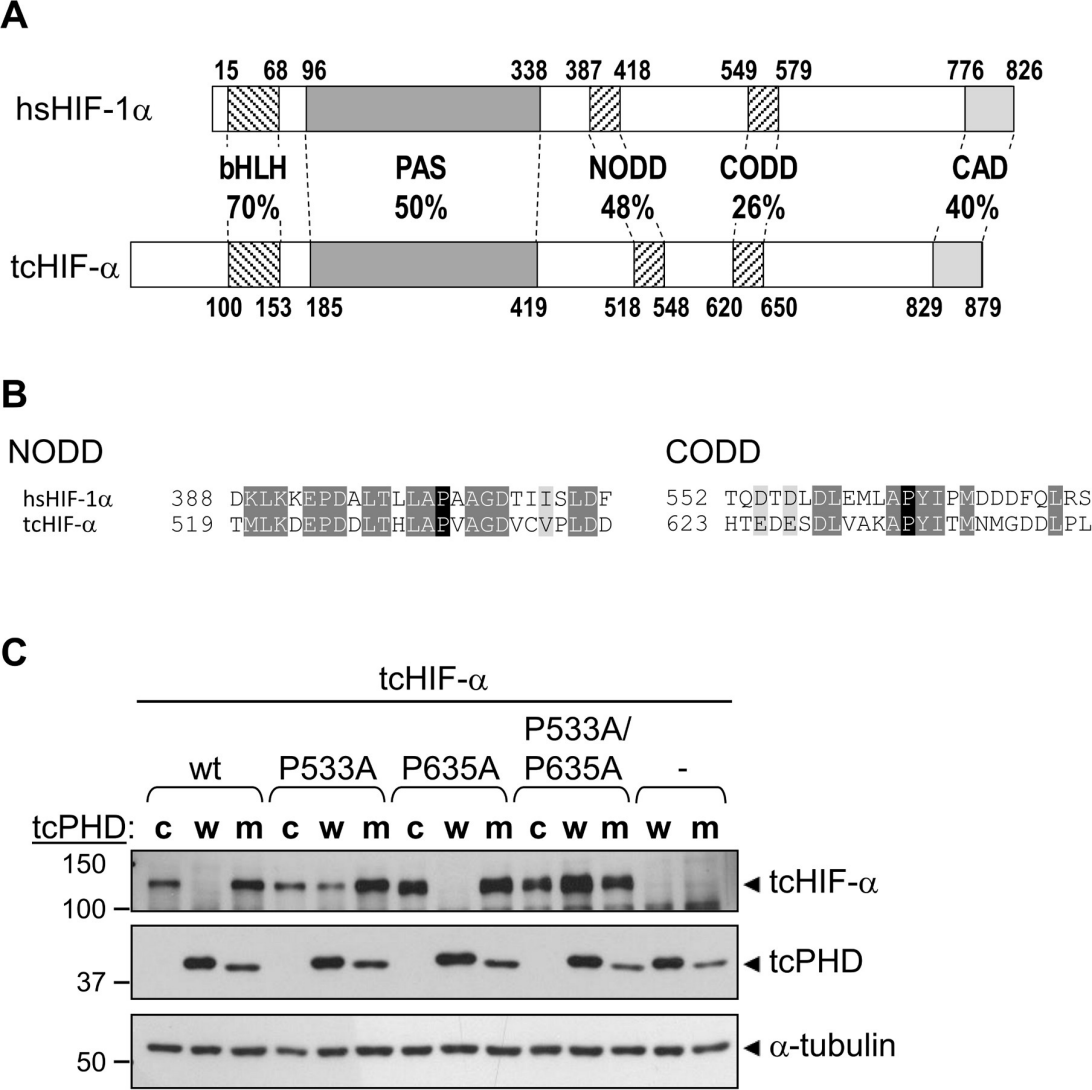
T. castaneum PHD mediates degradation of T. castaneum HIF-α. (A) Comparative domain structure of hsHIF-1α and tcHIF-α showing the percent identity of amino acid sequence in conserved regions, including the basic helix loop helix (bHLH), Per ARNT Sim homology domain (PAS), N- and C-terminal oxygen-dependent degradation domains (NODD and CODD) and C-terminal activation domain (CAD). Amino acid numbers at the start and end of each domain are shown. (B) The NODD and CODD amino acid sequences from tcHIF-α are shown aligned with the equivalent domains from hsHIF-1α. Conserved and similar residues are indicated by dark and light grey highlights, respectively, and the hydroxylated prolines (Pro533 in the NODD, Pro635 in the CODD) are in black. Amino acid numbers are to the left of each sequence. (C) The ability of tcPHD to facilitate degradation of tcHIF-α was assessed in mammalian cells. pEF-IRES-myc-6His-Puro6 plasmids, either empty (“-“) or encoding wild-type (wt) tcHIF-α, tcHIF-α single proline to alanine mutants P533A or P635A, or tcHIF-α double mutant P533A/P635A were transiently transfected into HEK293T cells along with pcDNA3.1 encoding a V5 epitope tag and wild-type tcPHD (“w”), catalytic mutant tcPHD H321A (“m”) or non-specific control Aryl Hydrocarbon Receptor aa 84–287 (“c”). Cells were then incubated for 8 hrs in normoxia. Levels of tcHIF-α and tcPHD protein in cell extracts were subsequently analysed by western blotting for myc and V5 epitope tags, respectively. α-tubulin served as a loading control.

Prior to examining FIH and the HIF-α CAD, initial experiments with *T*. *castaneum* examined the more widely conserved feature of HIF-α homologs, the ODD, as PHD-mediated hydroxylation of the ODD and consequent regulation of HIF-α stability is common to all HIF-α homologs characterised to date [8, 23, 34]. Firstly, the *T*. *castaneum* HIF-α homolog (tcHIF-α) was assessed for amino acid sequence conservation of the NODD and CODD regions compared with hsHIF-1α. HMMER searches for both domains returned a single hit for each, with the NODD showing greater sequence identity with hsHIF-1α (48%) compared to the CODD (26%) (Fig 6A and 6B). Both domains, however, contain key proline residues that are the likely target of prolyl hydroxylation (highlighted in black in Fig 6B). To find the hydroxylase(s) which may modify these prolyl residues, tBLASTn searches of the *T*. *castaneum* genome were performed using human PHD1, 2 and 3 protein sequences. A single candidate was identified which displayed high similarity to human PHD2 (59% amino acid sequence identity within the C-terminal catalytic domain (aa 181–426)). Importantly, the conserved residues in this putative PHD included those involved in binding of iron and the PHD cofactor, 2-OG (S1 Fig) [53].

A key question was whether tcHIF-α is regulated by tcPHD. As PHD enzymes are typically difficult to express and purify from *E*. *coli*, rather than using an in vitro hydroxylation assay, PHD activity was analysed in a cell-based assay through the enzyme’s ability to degrade HIF-α protein. The tcHIF-α and tcPHD sequences were cloned into expression vectors with myc and V5 epitope tags, respectively, and transiently transfected into HEK-293T cells. In the absence of any tcPHD co-transfection, anti-myc immunoblotting clearly demonstrated the presence of a specific tcHIF-α band migrating at approximately 130 kDa, 30 kD greater than the predicted size of 100 kD (lane 1 in Fig 6C). This is consistent with the higher apparent molecular weight of hsHIF-1α (approximately 120 kD compared with 90 kD predicted), and suggests that the tcHIF-α is post-translationally modified in a similar manner to mammalian HIF-1α.

Notably, tcHIF-α was easily detectable on the western blot, despite the use of normoxic conditions during the experiment. This has been observed previously with hsHIF-α, where over-expression saturates the ability of the PHD/VHL system to efficiently degrade hsHIF-α in normoxia, but this is alleviated by over-expression of VHL or PHDs [54–56]. Therefore, if tcHIF-α is targeted by tcPHD, then simultaneous over-expression of tcPHD should increase the efficiency of tcHIF-α degradation. In agreement, over-expression of V5-tagged tcPHD reduced tcHIF-α protein to undetectable levels in normoxia, confirming that tcHIF-α is regulated by tcPHD (Fig 6C lane 2). To verify that this repressive effect was dependent upon tcPHD catalytic activity, an iron binding-defective mutant of tcPHD (tcPHD H321A) was also cloned and tested. Surprisingly, while the change of just a single amino acid was confirmed by sequencing, the migration of the mutant protein was visibly faster than the wild type protein in the western blot (Fig 6C, lanes 2 and 3), for reasons that are not clear. Importantly, in contrast to wild-type enzyme, over-expression of this mutant failed to repress tcHIF-α levels (Fig 6C, lane 3), consistent with degradation mediated by the prolyl hydroxylase activity of tcPHD. To provide supporting evidence that tcHIF-α degradation is mediated by prolyl hydroxylation of the NODD and/or CODD, the tcHIF-α Pro to Ala mutants P533A, P635A and P533A/P635A were also examined. The P533A mutation had a clear stabilising effect on the tcHIF-α protein in the presence of tcPHD compared to wild type tcHIF-α (Fig 6C, lane 2 compared with lane 5), consistent with Pro533 being a target of tcPHD. While there is no obvious stabilisation of the P635A mutant compared to wild type tcHIF-α (Fig 6C, lane 8), the P533A/P635A tcHIF-α double mutant was completely unaffected by tcPHD over-expression (Fig 6C, lane 11). These results suggest that Pro533 is the major site of hydroxylation, with Pro635 likely being hydroxylated less efficiently and “fine-tuning” hydroxy-Pro533-mediated degradation. In summary, these data are consistent with tcHIF-α stability being regulated by prolyl hydroxylation by tcPHD in a similar manner to mammalian HIF-α proteins.

**Fig 7:**
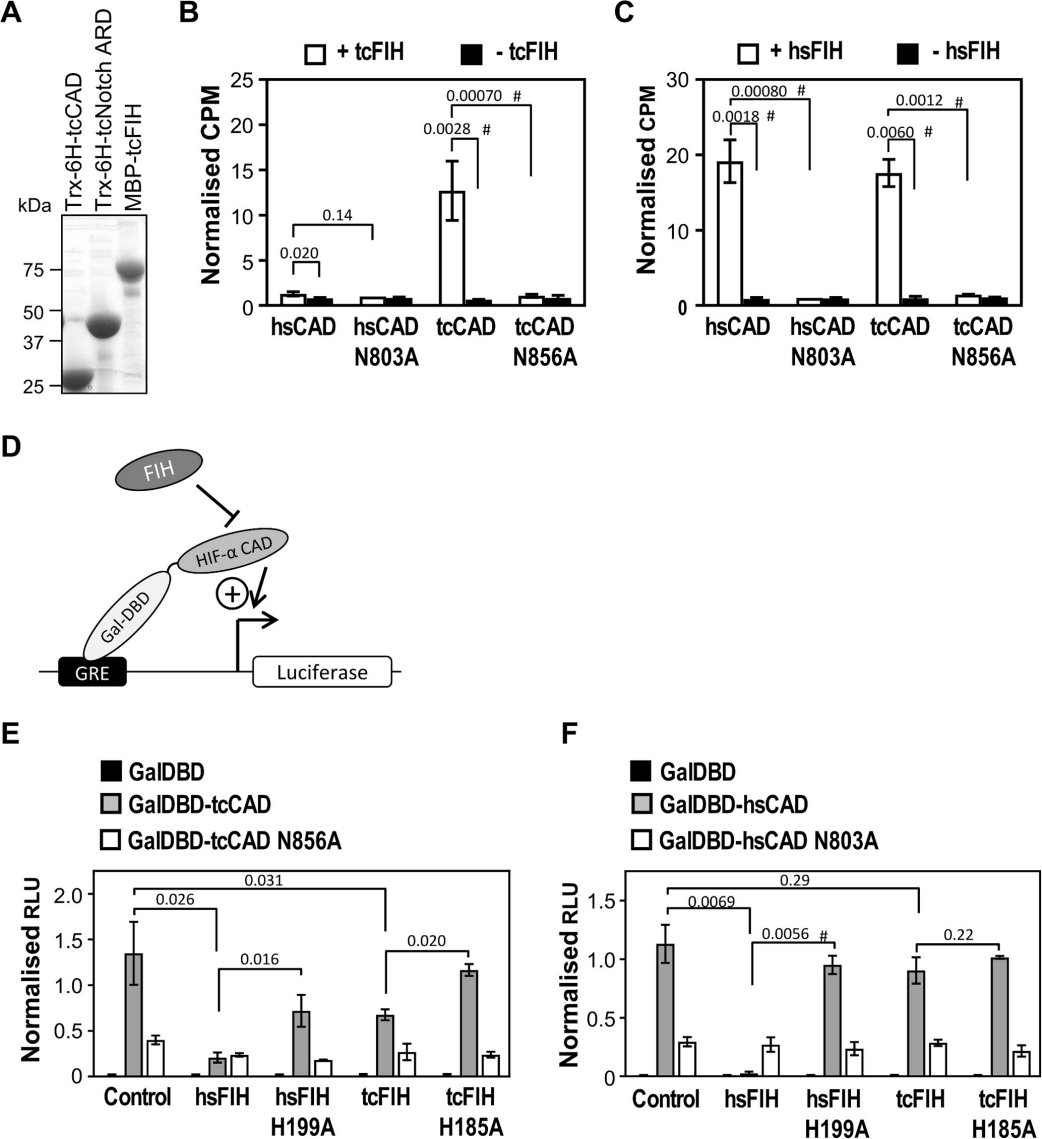
T. castaneum FIH is a putative asparaginyl hydroxylase that hydroxylates HIF-α substrates. (A) Thioredoxin-6 Histidine-tagged-tcHIF-α (790–879) (Trx-6H-tcCAD), Trx-6H-tcNotch (1747–1989) (Trx-6H-tcNotch ARD) and maltose binding protein-tagged tcFIH (MBP-tcFIH) were expressed in E. coli, purified by Ni^2+^ affinity or amylose agarose chromatography, and analysed by Coomassie-stained SDS-PAGE. (B) In vitro hydroxylation reactions were set up containing 60 μM tcHIF-α CAD (tcHIF-α residues 790–879), hsHIF-1α CAD (hsHIF-1α residues 736–826) and their corresponding Asn mutants, and either 1 μM tcFIH (white bars, analysed in triplicate) or buffer (black bars, analysed in duplicate). Reactions were incubated at 37°C degrees for 30 min, and then the counts per minute (CPM) of [^14^C]CO_2_ released during the reaction analysed by scintillation counting. Data were normalised to CPM observed for the hsHIF-1α CAD N803A + FIH sample. Bars are mean +/- SEM of combined data from 3 independent experiments. (C) As for part B, but using hsFIH enzyme. (D) Schematic representation of the reporter gene assay used to test transactivation capacity of tcHIF-α CAD. A firefly luciferase reporter gene downstream of a Gal4 response element (GRE) is transfected into mammalian cells. The GRE facilitates recruitment of yeast Gal4-DNA-binding domain (GalDBD)-tagged HIF-α CAD proximal to the luciferase gene promoter, with the activity of the CAD driving transcription. FIH-dependent HIF-1α CAD hydroxylation represses activity of the CAD, reducing transcription of luciferase. (E) Reporter gene assay testing tcHIF-α CAD transactivation potential, and the ability of hsFIH and tcFIH to repress CAD-mediated firefly luciferase production. FIH^-/-^ mouse embryonic fibroblasts (MEFs) were transiently transfected in triplicate with pGal-O-tcHIF-α CAD (GalDBD-tcCAD), pGal-O-tcHIF-α CAD N856A (GalDBD-tcCAD N856A) or empty vector (GalDBD), together with firefly and control renilla luciferase reporter constructs. Each well was also transfected with hsFIH or tcFIH, their catalytically inactive mutants (H199A and H185A, respectively), or empty pcDNA3.1 vector (“Control”). Relative luciferase units (RLU) were calculated from the ratio of firefly to renilla luminescence. Data were normalised to the average RLU from the GalDBD-tcCAD samples (with the exception of the sample co-expressed with hsFIH due to its small magnitude). Bars are mean +/- SEM of combined data from 3 independent experiments. (F) As for part E, except using GalDBD-hsHIF-1α CAD and GalDBD-hsHIF-1α CAD N803A in place of GalDBD-tcHIF-α CAD and GalDBD-tcHIF-α CAD N856A. Statistical analysis for parts B, C, E and F was carried out on non-normalised, log-transformed data using a 2-tailed paired t-test, with p values indicated above the bars. p values < 0.05 are considered significant. # indicates comparison also significant using the conservative Bonferroni-adjusted significance value of 0.0125 (for parts A and B) and 0.00625 (for parts E and F) for multiple comparisons.

To examine the ability of tcFIH to hydroxylate the tcHIF-α CAD, first an in vitro hydroxylation assay based on the release of [^14^C]CO_2_ from [^14^C] 2-oxoglutarate was performed, as FIH and the HIF-α CAD are amenable to recombinant expression in *E*.*coli*. The genes encoding tcFIH and *T*. *castaneum* HIF-α (residues 790–879) (tcHIF-α CAD) were cloned into expression vectors with MBP and thioredoxin-6 histidine tags, respectively. The tcFIH enzyme and tcHIF-α CAD substrate were expressed in *E*. *coli* along with their human counterparts, purified (Fig 7A), and then purified protein examined using the in the vitro hydroxylation assay. Reactions containing tcFIH and wild-type tcHIF-α CAD showed a large increase in [^14^C]CO_2_ released compared to background (Fig 7B), indicative of efficient hydroxylation, and consistent with tcFIH functioning analogously to hsFIH. However, tcFIH showed little activity when combined with wild-type hsHIF-1α CAD (Fig 7B). This observation implies that tcFIH and hsFIH have diverged structurally in order to efficiently hydroxylate their own CAD substrates. However, hsFIH was able to hydroxylate tcHIF-α CAD with a similar efficacy to hsHIF-α CAD (Fig 7C), suggesting that hsFIH has a more flexible substrate-binding capacity than tcFIH. Most importantly, these results indicate that a high degree of functional conservation exists between the human and *T*. *castaneum* HIF-α CAD substrates.

To confirm the location of FIH-dependent hydroxylation in the tcHIF-α CAD, site directed mutagenesis was used to mutate the predicted hydroxy-acceptor residue, Asn856, to Ala. Subsequent analysis of purified protein by in vitro hydroxylation assay indicated that the tcHIF-α CAD N856A mutant displayed negligible activity as a substrate with either enzyme (Fig 7B and 7C). Hence, like the hsHIF-1α CAD, this is consistent with a conserved single site of hydroxylation in the tcHIF-α CAD.

The investigation of tcHIF-α CAD as a substrate of tcFIH was extended to a cellular context. Since asparaginyl hydroxylation of the hsHIF-1α CAD modulates its binding to CBP/p300 coactivators, CAD transcriptional activity is a functional measure of FIH-dependent CAD hydroxylation. Hence a reporter gene assay was employed (modified from [4]), wherein HIF-1α CAD transactivation is monitored by generating a Gal4 DNA-binding domain/CAD (GalDBD-CAD) fusion protein and measuring its ability to drive a luciferase reporter gene (represented schematically in Fig 7D). FIH^-/-^ mouse embryonic fibroblast cells (MEFs) were transfected with plasmids encoding wild-type or Asn-mutant GalDBD-CAD, wild-type or catalytically inactive FIH, and luciferase reporter genes, incubated for 24 hrs, and relative luciferase levels measured.

Analysis of the results shows that firstly, in the absence of hsFIH or tcFIH over-expression, tcHIF-α CAD induced firefly luciferase expression well above basal levels (Fig 7E). This confirms that the tcHIF-α CAD functions as a transcriptional activator even in the mammalian cell context. The results also show that hsFIH very efficiently repressed the activity of hsHIF-1α CAD, as previously reported [3, 4], while impairment of hydroxylation, either through use of catalytically inactive FIH H199A or “hydroxylation-refractory” hsCAD N803A, greatly reduces this effect (Fig 7F). The repressive effect of hsFIH was also found to extend to tcHIF-α CAD, wherein hsFIH over-expression reduced its activity by almost 6.5-fold, compared to only 1.7-fold for the tcHIF-α CAD N856A mutant (Fig 7E). Significantly, tcFIH over-expression was also able to reduce the activity of tcHIF-α CAD, although not as efficiently as hsFIH. This effect is likely to be at least partially dependent upon hydroxylation of the tcHIF-α CAD, as use of tcHIF-α CAD N856A or catalytically inactive tcFIH H185A reduced the magnitude of the repression (Fig 7E). Lastly, it was observed that tcFIH had little effect on the activity of the hsHIF-1α CAD, consistent with the inability of tcFIH to hydroxylate hsHIF-1α CAD in vitro (Fig 7F). Taken together, these data show modulation of tcHIF-α CAD activity by tcFIH-dependent hydroxylation in a cellular context, and demonstrate the conservation of FIH-dependent HIF-α CAD regulation between *T*. *castaneum* and higher vertebrates.

### Functional conservation of ARD hydroxylation in *T*. *castaneum*

**Fig 8:**
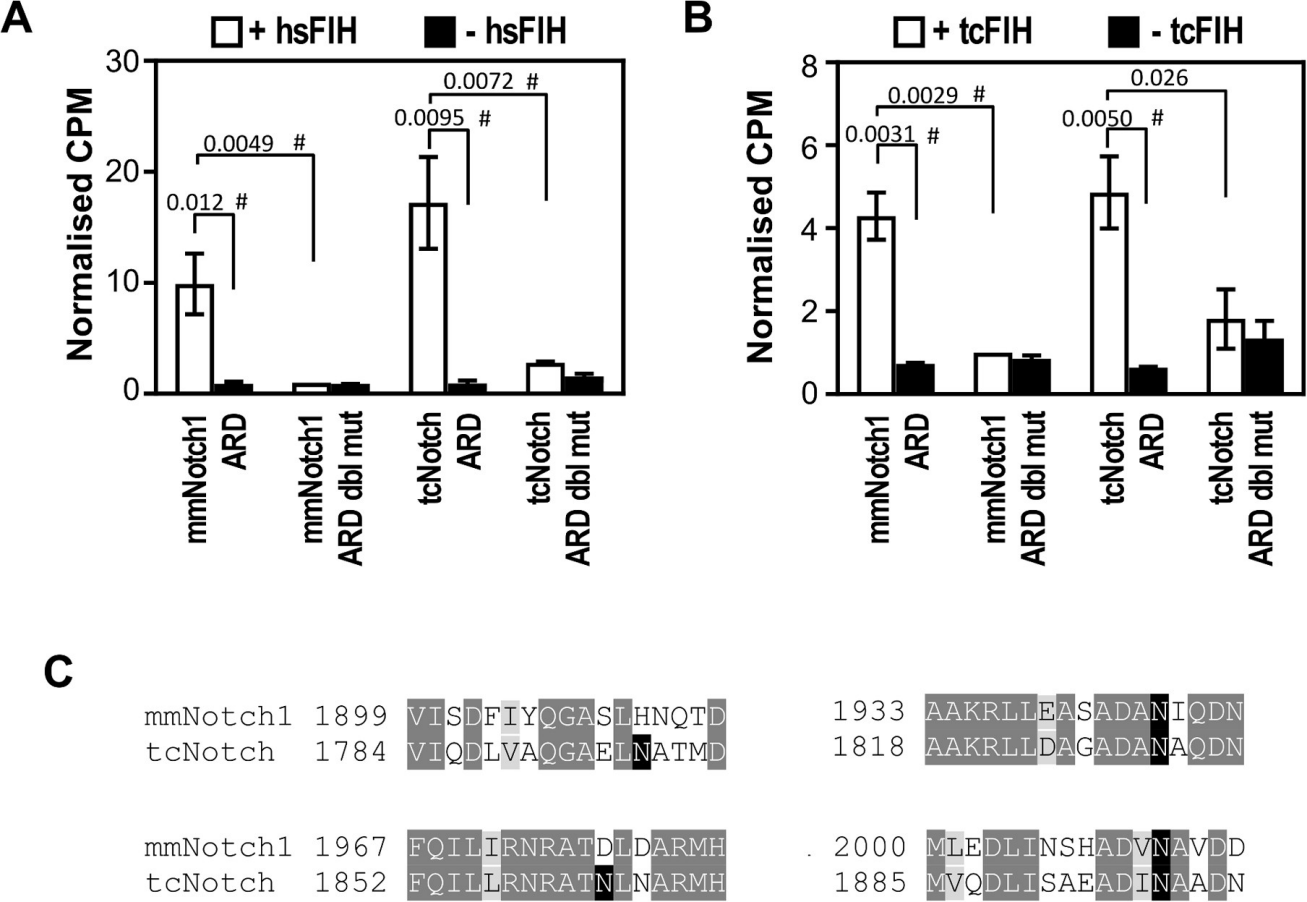
Hydroxylation of ARD-containing substrates by T. castaneum FIH. (A) 1 μM MBP-tagged tcFIH was tested in triplicate in the presence of 40 μM Trx-6H-tagged mmNotch1 (1862–2104) (mmNotch1 ARD) or Trx-6H-tcNotch (1747–1989) (tcNotch ARD) and their Asn to Ala mutants mmNotch1 ARD NN1945/2012AA (mmNotch1 ARD dbl mut) and tcNotch ARD NN1830/1897AA (tcNotch ARD dbl mut) by in vitro hydroxylation assay (white bars). As a control, each substrate was also tested in duplicate with buffer in place of enzyme (black bars). Data from each experiment were normalised to CPM (counts per minute of [^14^C]CO_2_ released) for the mmNotch1 ARD dbl mut + FIH sample. Bars are mean +/- SEM of combined data from 3 independent experiments. (B) As for (A), but using hsFIH. (C) Alignment of sequences within tcNotch predicted to contain target Asns with their equivalent regions in mmNotch1. Conserved and similar residues are indicated by dark and light grey highlights, respectively, and the (putative) target Asns are in black. Amino acid numbers are to the left of each sequence. Statistical analysis for parts A and B was carried out on non-normalised, log-transformed data using a 2-tailed paired t-test, with p values indicated above the bars. p values < 0.05 are considered significant. # indicates comparison also significant using the conservative Bonferroni-adjusted significance value of 0.0125 (for parts A and B) for multiple comparisons.

In mammals, CAD and ARD hydroxylation by FIH coexist, which may represent independent regulatory activities of FIH, and/or a requirement for “ARD pool” mediated adjustment of HIF-1α CAD transactivation. To ascertain if ARD hydroxylation exists alongside CAD hydroxylation in *T*. *castaneum*, we first analysed the ability of tcFIH to hydroxylate a well-characterised ARD-containing substrate of FIH, Notch1. Wild-type Trx-6H-Notch1 (*Mus musculus* 1862–2104) (mmNotch1 ARD) or a mutant wherein the two Asns targeted by FIH were mutated to Ala (mmNotch1 ARD NN1945/2012AA) were expressed in *E*. *coli*, purified, and tested by in vitro hydroxylation assay with tcFIH and hsFIH. Control reactions containing hsFIH with mmNotch1 ARD showed high levels of [^14^C]CO_2_ released compared to background (Fig 8A), indicative of efficient hydroxylation, as has been shown previously [12, 17]. Importantly, a similar strong release of [^14^C]CO_2_ was also seen with when tcFIH was used in place of hsFIH (Fig 8B), again demonstrating the strong functional conservation between these two enzymes, but here with an ARD substrate. Moreover, it is likely that this functional conservation extends to the particular Asns targeted for hydroxylation, as use of mmNotch1 ARD NN1945/2012AA as a substrate abolished the activity of both hsFIH and tcFIH (Fig 8A and 8B).

Next, it was sought to establish whether ARD hydroxylation by tcFIH extended to a *T*. *castaneum* Notch substrate. BLAST searches using the human Notch1 sequence revealed a protein with 46% amino acid identity to Notch1 (tcNotch). Importantly, closer inspection of tcNotch’s ARD region revealed the presence of four putative FIH target sites with varying similarity to hsFIH’s preferred target sequence (Fig 8C). To test if these sites were targeted by hsFIH or tcFIH, tcNotch 1747–1989 (tcNotch ARD) was cloned, expressed, purified (Fig 7A) and examined as a substrate by in vitro hydroxylation assay. In the presence of hsFIH, tcNotch ARD was found to stimulate strong 2-OG decarboxylation, consistent with this enzyme’s flexible ARD hydroxylation ability (Fig 8A). Interestingly, tcFIH also displayed robust activity in the presence of tcNotch ARD. Furthermore, the hydroxylation specificity of tcNotch ARD by both FIHs is conserved, with activity of either hsFIH or tcFIH reduced to near background levels by alanine mutagenesis of just two of the four putative hydroxylation sites in tcNotch, Asn1830 and Asn1897, corresponding to the two target sites in mmNotch1 (Fig 8A and 8B). Overall, it can be concluded that Notch (and hence ARD) hydroxylation is functionally conserved between vertebrates and *T*. *castaneum*, as is its coexistence with FIH-mediated HIF-α CAD hydroxylation. It is worth noting, however, that unlike the results obtained in the HIF-α CAD hydroxylation assays, the efficiency of cross-species Notch hydroxylation was comparable for both beetle and human FIH. Although the mechanism for this change in substrate specificity remains to be elucidated, it is interesting that tcFIH’s CAD hydroxylation abilities are more restricted, while Notch ARD hydroxylation remains unaffected between FIH enzymes from these diverse species.

### FIH functionality in the coral, *Acropora millepora*

**Fig 9:**
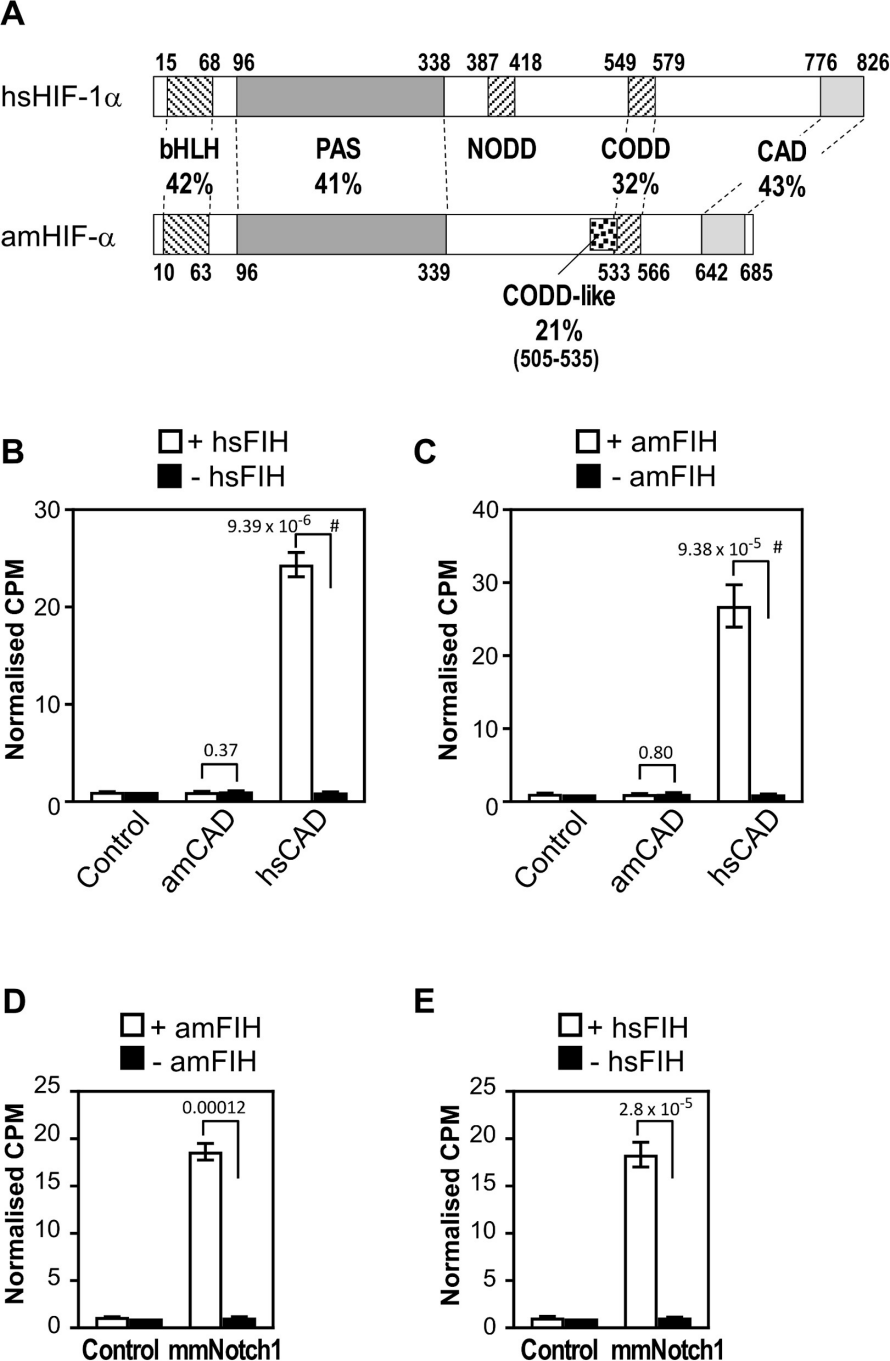
Hydroxylation of CAD and ARD substrates by A. millepora FIH. (A) Comparative domain structure of hsHIF-1α and A. millepora HIF-α (amHIF-α) showing the percent identity of conserved regions, including the basic helix loop helix (bHLH), Per ARNT Sim homology domain (PAS), C-terminal oxygen-dependent degradation domain (CODD) and C-terminal activation domain (CAD). Unlike human and T. castaneum HIF-α, amHIF-α does not appear to contain a NODD, but instead contains a second sequence with low similarity to the human CODD (“CODD-like”) just N-terminal of a more robustly conserved CODD sequence. Amino acid numbers at the start and end of each domain are shown. (B) MBP-tagged hsFIH (1 μM) was tested in triplicate by in vitro hydroxylation assay with 25 μM Trx-6H-tagged hsHIF-α CAD and amHIF-α CAD substrates (hsHIF-1α (736–826) and amHIF-α (604–693), respectively), or buffer alone control. (C) As for part B, except using 1 μM MBP-tagged A. millepora FIH (amFIH). (D) MBP-tagged amFIH (at 1 μM) was tested in triplicate with mouse Trx-6H-Notch1 (1862–2104) (mmNotch1, 25 μM) or buffer alone (control) by in vitro hydroxylation assay. (E) As for D, but using hsFIH. For parts B-E, data from each experiment were normalised to CPM (counts per minute of [^14^C]CO_2_ released during the reaction) observed for the control minus FIH sample. Bars are mean +/- SEM of combined data from 3 independent experiments. Statistical analysis was carried out on non-normalised data using a 2-tailed paired t-test, with p values indicated above the bars. p values < 0.05 are considered significant. For parts B and C, # indicates comparison also significant using the conservative Bonferroni-adjusted significance value of 0.025 for multiple comparisons.

Although HIF-α CAD hydroxylation is conserved in *T*. *castaneum*, our analysis of invertebrate HIF-α homologs showed that many have “imperfect” FIH preferred target sequences (purple species from Fig 3). The -8 Leu relative to the target Asn was the most frequently divergent residue, although variations in the -1 Val and -2 acidic residue were also occasionally observed (Fig 4). While the importance of the -8 Leu is currently unknown, alanine scanning mutagenesis experiments imply that the -1 Val (but not the -2 acidic residue) is important for catalysis by hsFIH [47]. Consequently, if other FIH homologs replicate hsFIH substrate specificity, species with imperfect HIF-α CAD target sequences may have evolved a reduced-efficiency of hydroxylation of the CAD by FIH. Alternatively, these target site “imperfections” may be accommodated by differences in the relevant species’ FIH homolog, thus preserving CAD hydroxylation. To assess these possibilities, the hydroxylation of the HIF-α CAD by FIH in *Acropora millepora* (stony coral) was examined. This choice was motivated not only by *A*. *millepora* having an imperfect target sequence (IXXXXXEVN), but also because this species is amongst the simplest organisms to contain both candidate FIH and a clear CAD-containing HIF-α homolog (depicted schematically in Fig 9A).

Full-length *A*. *millepora* FIH and the sequence encoding the final 90 amino acids of *A*. *millepora* HIF-α (amFIH and amHIF-α CAD, respectively) were cloned, expressed and purified from *E*. *coli* along with their human counterparts, and tested by in vitro hydroxylation assay. Although imperfect target sequences in ARDs can be hydroxylated by hsFIH in vitro (see e.g. [11, 16]), combining amHIF-α CAD with hsFIH did not stimulate activity above the background seen with hsFIH alone (Fig 9B). Surprisingly, the same results were seen when similar experiments were performed with amFIH (Fig 9C). In contrast, amFIH and hsFIH both promoted robust 2-OG decarboxylation when in the presence of hsHIF-1α CAD (Fig 9B and 9C), demonstrating that, despite the large evolutionary distance between them, these enzymes share considerable functional homology. Furthermore, while it cannot be ruled out that FIH-mediated hydroxylation of coral CAD requires an in vivo setting, these data suggest that the CAD of amHIF-α is not regulated by FIH in an analogous manner to its human counterpart.

A possible explanation for amFIH’s inability to hydroxylate amHIF-α CAD is that amFIH has alternative roles in this species, such as hydroxylation of ARD-containing proteins. To examine the ability of amFIH to target the ARD structural motif, the enzyme was tested by in vitro hydroxylation assay with Trx-6His-tagged mmNotch1 ARD substrate. As is seen with the human homolog of FIH, combination of amFIH with mmNotch1 ARD resulted in high activity (Fig 9D and 9E). Thus, these data support the idea that ARD targeting is a highly conserved feature of both amFIH and hsFIH. More importantly, these data, together with the observed absence of amHIF-α CAD hydroxylation, raise the possibility that an ARD-containing protein may be the primary target of FIH in coral. Extending this idea, we hypothesised that amFIH’s apparent preference for ARD hydroxylation reflects the ancestral, “pre-HIF-α” function of the FIH enzyme. However, preliminary hydroxylation assays testing the premetazoan FIH candidates from *S*. *rosetta* and *C*. *owczarzaki* argue against this: mmNotch1 ARD substrate showed no 2-OG decarboxylation with *C*. *owczarzaki* FIH, and activity only barely above background with *S*. *rosetta* FIH (S2A–S2C Fig). Assays using hsHIF-1α CAD as a substrate showed a similar lack of robust activity (S2D–S2F Fig). While it cannot be ruled out that these results are a reflection of poor protein folding of the recombinantly expressed enzymes, the use of sub-optimal assay conditions or inaccurate prediction of enzyme sequences, it may indicate that the ARD target sequence preferences for FIH in premetazoans differ to those in vertebrates. Alternatively, FIH homologs in basal species may have an entirely different primary substrate.

## Discussion

### CAD regulation by FIH

The detailed analysis of FIH and HIF-α CAD homologs presented herein has shown that a number of factors likely shape their conservation and functionality. While it is likely that FIH has HIF-α CAD-independent roles in certain organisms (e.g. in premetazoans or CAD^—^FIH^+^ species), it is clear that numerous species co-conserve FIH and a FIH target motif-containing HIF-α CAD. This suggests that HIF-α CAD regulation by FIH is an important feature of HIF-α biology both within and beyond vertebrates. Such an assumption is supported by our examination of the *T*. *castaneum* HIF-α/FIH system, which demonstrated that tcHIF-α CAD is a tcFIH substrate in vitro, and that expression of tcFIH in mammalian cells could repress tcHIF-α CAD activity. Moreover, the similarity to the mammalian HIF-α system is perpetuated by tcPHD, the expression of which destabilised the tcHIF-α homolog. Indeed, the activity of *T*. *castaneum* HIF-α, FIH and PHD within mammalian cells is itself a further testament to their functional conservation, since interaction with human homologs of transcriptional cofactors or degradation machinery would be required to achieve these regulatory effects.

In contrast to the *T*. *castaneum* data, and despite its conservation of a target Asn, amHIF-α CAD was not a substrate of amFIH or hsFIH in the in vitro hydroxylation assay. This result is at odds with a report showing that a HIF-α CAD peptide derived from fellow cnidarian, *Nematostella vectensis* (sea anemone), was a substrate of hsFIH, although the efficiency of the modification was not reported [23]. A poor targeting efficiency could be explained by the FIH target motif in amCAD diverging from that preferred by hsFIH at the -8 position (a feature which is observed in many species (Fig 4)), replacing Leu with Ile. While the specific contribution of this residue was not investigated, our data indicate that amFIH efficiently targets the hsHIF-1α CAD. The C-terminus of hsHIF-1α CAD diverges from amHIF-α CAD at only a small number of positions, including possession of a -8 Leu (S3 Fig), implicating Leu at this position as necessary for efficient hydroxylation. Furthermore, if poor hydroxylation is a genuine, evolutionarily enforced feature of stony coral HIF-α CADs, a logical prediction would be the universal absence of a -8 Leu in these species. This is not the case, although the one species (*Pseudodiploria strigosa*) which did contain a -8 Leu diverged at a different hsFIH target motif residue, namely -1 Val to Met (S3 Fig). It is intriguing to speculate that variation of this residue (believed to strongly influence hsFIH’s catalytic rate [47]) may likewise render *P*. *strigosa* HIF-α CAD a poor FIH substrate.

Although co-existence of a non-targeted HIF-α CAD with FIH may seem counterintuitive (and indeed, it cannot be ruled out that the poor in vitro hydroxylation of amHIF-α CAD does not represent the in vivo situation), there are several possible explanations. The kinetics of FIH’s interaction with the HIF-α CAD will mainly affect two parameters: (1) the CAD’s sensitivity to oxygen levels, and (2) FIH’s availability to hydroxylate/bind other proteins. Regarding the first point, a lack of HIF-α CAD hydroxylation does not preclude FIH-CAD complex formation, which could in turn affect CAD transactivation via competition with CBP. Importantly, this process may still be oxygen-sensitive, as all metazoan FIH homologs tested thus far can hydroxylate mmNotch1 (and presumably other ARD-containing proteins). This implies that the “ARD sink effect” (i.e. unhydroxylated ARDs mopping up free FIH during periods of hypoxia) could also exist in other species. However, given that the verified target Asn-containing non-substrate of hsFIH, mmNotch4, has significantly reduced binding affinity relative to substrate mmNotch1 [48], a competitive binding hypothesis should be treated with caution. Alternatively, a poorly targeted HIF-α CAD may be constitutively active. The existence of HIF-α CAD-like sequences in numerous FIH-lacking species supports (provided that these CAD-like sequences retain CBP binding capacity) the concept of constitutive CAD domains. However, if such a HIF-α CAD exists within *A*. *millepora*, it seems curious that a “near perfect” hsFIH target motif would be conserved within this domain. Indeed, in many FIH deficient species, the FIH target motif has been likewise been lost from the HIF-α CAD. Nonetheless, it is possible that residues typically involved in FIH targeting are critical for CBP binding in certain organisms.

As a final consideration, it is possible thatHIF-α CAD sequences are a direct result of the availability of FIH for hydroxylation. This in turn may be dictated by the number and binding/hydroxylation efficiency of additional FIH interactors within the cell.

### Other FIH substrates/interacting partners

The search for HIF-α-independent FIH roles with functional outcomes in mammals has been underway for over a decade, and has proven a difficult task. However, the identification of putative FIH homologs in premetazoans which lack HIF-α is the strongest evidence yet that, irrespective of its role in vertebrates, FIH did not evolve solely for HIF-α modulation. The nature of FIH’s role in premetazoans remains to be determined. Indeed, our preliminary analysis of premetazoan FIH substrate specificity infers that mammalian HIF-α and Notch proteins are, at best, only very poorly targeted. In turn, this may indicate that some premetazoan FIHs are catalytically inactive, have different ARD target sequence preferences to those found in mmNotch1, or have a completely different substrate altogether. Fortuitously, the comparatively simplistic biology of premetazoans could help to better define FIH’s role in basal eukaryotes, particularly in light of rapidly advancing CRISPR/Cas technologies [57] which may facilitate the creation and analysis of FIH null organisms.

Similar to the premetazoan species which lack HIF-α, a small number of Metazoa were identified which conserved a FIH homolog, despite possessing a CAD^—^or target Asn-deficient HIF-α protein. Again, this points to a HIF-α-independent functional role for FIH in these species, although the nature of this role remains to be identified. One possibility is that FIH’s functional partner in premetazoans has a homolog in Metazoa. Naturally, conservation of a premetazoan-derived functional partner does not preclude the evolution of novel substrates or interactors in higher order species. Likewise, whether such additional partners would create pressure for a change in the FIH target sequence in the HIF-α CAD (i.e. whether CAD sequences can be used to predict substrate repertoires within species) will require a more in depth understanding of FIH’s catalysis and interaction kinetics.

## Conclusions

The work presented here is the first demonstration of catalytically active FIH homologs beyond the Vertebrata. While the FIH enzyme regulating tcHIF-α is part of a system with strong similarity to the human HIF-1α/FIH/PHD axis, the FIH homolog in the coral, *A*. *millepora*, did not appear to modify its corresponding amHIF-α CAD homolog, despite effectively hydroxylating mammalian HIF-1α CAD/Notch ARD substrates. This finding, combined with the intriguing identification of FIH^+^CAD^—^species is further evidence for the existence of HIF-α-independent roles for FIH. The nature of these roles, specifically whether they include ARD hydroxylation, how and when they coexist with HIF-α CAD regulation, and their relation to the metabolic function of FIH in mammals are important topics for future study.

## Materials and methods

### *T*. *castaneum* strains

The beetles used in this study were either QTC4 or QTC931 strains, and were a kind gift from Pat Collins (Department of Agriculture and Fisheries, Brisbane, Australia). Beetles were cultured at room temperature and humidity in organic barley flour.

### *T*. *castaneum* RNA extraction and cDNA synthesis

Total RNA was isolated from whole insects from pupal to adult stages by using the RNeasy Mini Kit (Qiagen) as per the manufacturer’s instructions. 2 μg of total RNA was then used as a template for cDNA synthesis using 300 ng random hexamer and 500 ng poly-dT primers, and Superscript III (Invitrogen).

### Identification of species homologs of FIH, HIF-α, Notch and PHD

Initial sequence searching of NCBI (WGS, TSA, EST and nr protein/nucleotide) and Ensembl databases was performed using human protein homologs of FIH, HIF-α (either full length or the final 46 aa only), Notch and PHDs 1–3, and the pBLAST or tBLASTn algorithms [43] (see S1 Table for sequence accessions and BLAST E value scores). Likely homologs were selected using various methods. For FIH, BLAST hits with an alignment score greater than 200 bits were examined manually for conservation of hsFIH residues His199, Asp201, Arg238, Gln239 and His279. Hits were also required to contain two out of the three 2-OG-binding residues Tyr145, Thr196 and Lys214 to be classified as FIH. When searching for homologs of HIF-α and its CAD domain, it was found that BLAST searches using full-length hsHIF-1α and default parameters could regularly locate the bHLH-PAS domain from putative HIF-1α homologs, but were less capable of detecting the NODD, CODD or CAD (regions of approximately 40 aa in size either centred around Pro402, Pro564 or encompassing the C-terminus of HIF-1α, respectively). This is presumably due to the small size and partial conservation of residues in these domains. To more reliably locate these domains, translated genomic or transcriptomic BLAST partial hits were further searched using HMMER 3.0 [44] (with default parameters) and hidden markov models built from characterised and newly identified HIF-α homologs (which were further modified as new hits were located). The limited capabilities of BLAST were also problematic for locating 5’ truncated HIF-α sequences which lacked the full bHLH-PAS domain. For this reason, the C-terminal 46 aa of hsHIF-1α was also used to search sequence databases (sometimes prior to querying with full length HIF-1α), thus improving HIF-α identification ability by excluding often large stretches of non-conserved sequence adjacent to the CAD. Note that query sequences listed for each HIF-α hit in S1 Table are simply the first query sequences which produced a successful hit for follow-up, and do not necessarily indicate that a full length hsHIF-1α query was not similarly successful. A sequence was classified as encoding part or all of HIF-α if (1) BLAST/HMMER hits were located on a single sequence or numerically neighbouring genomic contigs, (2) the sequence(s) contained either a full (or partial if the sequence was 5’ truncated) match to the bHLH-PAS domain, (3) at least one other domain among the NODD, CODD or CAD. A species was only classified as lacking the CAD if no CAD BLAST/HMMER hits were located and the species’ genome had been sequenced. Furthermore, to aid in identification of new homologs, searches in selected species groups were sometimes followed up by “nearest species neighbour” BLAST searches using newly identified hits from that group. Non-human query sequences were routinely subjected to reciprocal BLASTing against the human nr protein database to verify that hsHIF-1α, 2α or 3α was the top hit (S1 Table).

### Cladogram generation

A Newick-formatted cladogram was generated using phyloT (http://phylot.biobyte.de/), with settings “collapsed internal nodes” and “polytomy”. PhyloT displays species relationships based on those in NCBI taxonomy. Colouring and shading of species scientific name text was performed using Interactive Tree of Life (itol.embl.de/).

### Plasmids

For cloning of *T*. *castaneum* homologs, coding sequences were amplified from cDNA using primer pairs incorporating restriction sites (S2 Table). amFIH and amHIF-α were identified, cloned, and a kind gift from David Hayward (Australian National University, Australia).

PCR products were cloned into pET32a (Novagen) or pET32a destination vector (Trx-6H-tagged proteins), pGal-O [58] (tcHIF-α (790–879)), pMBP [59] (MBP-tagged enzymes), pcDNA3.1 (Thermofisher) (tcPHD and tcFIH), or pEF-IRES-myc-6His-Puro6 (pEF-IRES-Puro6 [60] modified with a myc-6His oligo)(tcHIF-α) either directly using the underlined restriction sites, or indirectly via pGEM-T Easy (Promega) and the same restriction sites. pET32a-TEV-hsFIH-NTKGVE/TTP was generated through multiple rounds of overlap extension PCR with template pET32a-TEV-FIH [60]. Plasmids pET32a-hsHIF-1α (737–826) and pET32a-hsHIF-1α (737–826) N803A [47], pMBP-hsFIH and pMBP-hsFIH H199A [3], pGal-O-hsHIF-1α (737–826) [47], pET32a-mmNotch1 (1862–2104) and pET32a-mmNotch1 (1862–2104) NN1945/2012AA [17], and pET32a-mmHIF-1α (747–836) [48], have been described elsewhere. pcDNA3.1-DR (84–287) was a kind gift from A. Chapman-Smith (Department of Molecular and Cellular Biology, University of Adelaide).

S. rosetta and C. owczarzaki FIH (as per the sequences referred to in S1 Table) were cloned into pMBP using Gibson assembly of gBlocks.

### Expression and purification of recombinant proteins

All Thioredoxin (Trx)-6His-tagged and maltose binding protein (MBP)-tagged proteins were expressed and purified as described previously [3, 60], with the exception of the Trx-6His-hsFIH and Trx-6His-tcFIH enzymes. Expression of these proteins was identical to those described above, but purification was carried out using the Profinia Protein Purification System (Bio-rad). Bacterial cell lysates were loaded onto a 1 mL Bio-scale Mini Profinity IMAC cartridge, washed with 6 column volumes (CV) of lysis buffer (20 mM Tris-HCl pH 8, 150 mM NaCl, 5 mM imidazole, 1 mM PMSF (added fresh), 0.5 mM DTT (added fresh)), 6 CV wash buffer (20 mM Tris-HCl pH 8, 150 mM NaCl, 10 mM imidazole), and then eluted in 3.5 CV elution buffer (20 mM Tris-HCl pH 8, 150 mM NaCl, 250 mM imidazole). 2.5 mL of eluate was then loaded onto a PD-10 column (GE) and buffer exchanged into protein storage buffer (20 mM Tris-HCl pH 8, 150 mM NaCl) as per the manufacturer’s instructions. Due to a tendency of Trx-6His-tcFIH to precipitate after short term storage at 4°C, purified protein was immediately diluted to approximately 10 μM after buffer exchange. Recombinant polypeptide concentrations were calculated using their extinction coefficients and absorbance at 280 nm, and purity was assessed by densitometry of Coomassie-stained SDS PAGE gels.

### Hydroxylation assays

FIH activity was assayed by a method based on the hydroxylation-coupled decarboxylation of 2-oxo[1-^14^C]glutarate, described in detail in [60]. Enzyme and substrate concentrations used for each assay are detailed in the figure legends. K_m_ values for hsFIH with hsHIF-1α CAD and mmNotch1 ARD are 40 μM and <0.2 μM, respectively. Thus, for simple testing of novel proteins substrates (as opposed to detailed kinetic analysis), it was endeavoured to use at least 60 μM CAD or 25 μM Notch substrate. This reduces the influence of dropping substrate concentrations (and therefore a reduction in FIH’s catalytic rate) on the total [^14^C]CO_2_ released during the reaction, without requiring that substrate proteins be concentrated prior to employment in the assay. Due to variable solubility of purified substrates, however, sometimes lower concentrations had to be used (see e.g. Fig 9).

### Cell culture and transient transfections

The human embryonic kidney 293T (HEK293T) cell line (a kind gift from K. Jensen, University of Adelaide, originally supplied by ATCC) and mouse embryonic fibroblast FIH knock out (FIH^-/-^ MEF) cell line [22] were grown in DMEM (Gibco) supplemented with 10% fetal calf serum. Cell lines were not assessed for mycoplasma contamination. Transient transfections of HEK293T and FIH^-/-^ MEF cells were performed using Lipofectamine 2000 (Invitrogen) and Fugene6 (Roche), respectively, according to the manufacturer’s instructions.

### Reporter assays

FIH^-/-^ MEFs were seeded at 35,000 cells per well in 24-well plates and allowed to grow for 18 hours before transfection in triplicate with relevant plasmids. Each well received 4 different constructs: (1) 100 ng of a GalDBD-CAD-encoding plasmid (pGal-O-hsHIF-1α (737–826) or pGal-O-tcHIF-α (790–879), with empty pGal-O used as a control, (2) 100 ng of a FIH-producing plasmid (pcDNA3.1-hsFIH, pcDNA3.1-hsFIH H199A, pcDNA3.1-tcFIH, or pcDNA3.1-tcFIH H185A, with pEF-BOS-CS used as a control [61], (3) 150 ng pGRE-luciferase [62], which encodes a Gal4 responsive element (GRE) upstream of a firefly luciferase gene, and (4) 10 ng pRL-TK (Promega), which encodes renilla luciferase downstream of a constitutive promoter. After 24 hours, cells extracts were prepared and analyzed by Dual luciferase reporter assay (Promega), wherein firefly luciferase levels are recorded and normalised to those of renilla luciferase, producing a “relative luciferase” measurement.

### *T*. *castaneum* HIF-α stability analysis

HEK293T cells were seeded in 6-well plates (at 20% confluency) and allowed to grow to 40% confluency before transfection with 2 μg of pcDNA3.1 containing either tcPHD, tcPHD H321A or Aryl Hydrocarbon Receptor (84–287), and 200 ng of pEF-IRES-myc-6His-Puro6 either empty or containing tcHIF-α, tcHIF-α P533A, tcHIF-α P635A, or tcHIF-α PP533/635AA. After 8 hours, cells were lysed in Laemmli buffer and analysed by western blot using anti-V5 antibodies for tcPHD, and anti-Myc for tcHIF-α.

### Immunoblotting assays

After separation by SDS PAGE on an 9% Tris Glycine gel, samples were blotted onto nitrocellulose filters (PALL BioTrace NT) and blocked in 10% w/v skim milk, 1% Tween 20 in phosphate-buffered saline (PBS). Filters were then incubated with primary antibodies: anti-Myc (1:2; mouse hybridoma supernatant 9E10), anti-V5 (1:15000, R960-25, Invitrogen), and anti-rat-α-tubulin (1:10000, YL1/2, Novus Biologicals) in PBS at 4°C O/N. Secondary antibodies (anti-mouse or anti-rat IgG-horseradish peroxidase conjugates (Amersham Pharmacia Biosciences)) were used at 1:20000 for 1 hr at room temperature in PBS. After washing, proteins were visualized using Immobilon Western Chemiluminescent HRP Substrate (Millipore) as per the manufacturer’s instructions.

### Statistical analyses

Triplicates in hydroxylation assay or reporter assay samples were assessed for normality using the Shapiro-Wilk test in SPSS, with the vast majority returning insignificant p values > 0.05. Two-tailed, paired t-tests were performed on non-normalised data, which were log-transformed to account for variations between biological replicates where indicated. Both “unadjusted” and Bonferroni-adjusted significance levels were reported for calculated p values, as suggested by [63].

## Supporting information

S1 Fig Alignment of human and *T*. *castaneum* PHDs. The three human HIF PHDs were aligned with *T*. *castaneum* PHD using Clustal Omega [51]. Residues strongly or partially conserved are shown in cyan and grey, respectively. Iron coordination (red) and 2-OG binding residues (dark blue) are also indicated. The structure of the catalytic domain of hsPHD2 is indicated above the alignment [53], with yellow arrows indicating the β-strands that comprise the DSBH.
(TIF)

S2 Fig Substrate specificity of putative premetazoan FIH homologs. (A-C) Assessment of MBP-tagged *Capsaspora owczarzaki* (coFIH) and *Salpingoeca rosetta* (srFIH) FIH homologs (at 1 μM each) by in vitro hydroxylation assay in the presence of 25 μM Trx-6H-mmNotch1 (1862–2104) (mmNotch1) substrate. Human FIH (hsFIH) served as a comparison for activity observed. Samples with (white and black bars) and without enzyme (grey bars) were tested in triplicate or duplicate, respectively. Bars are mean +/- SD. (D-F) As for A-C, but testing 25 μM Trx-6H-hsHIF-1α (736–826) (hsCAD) as a substrate. Data for parts A-F representative of 2 independent experiments.
(TIF)

S3 Fig Comparison of cnidarian CAD sequences. Predicted CAD sequences from a variety of cnidarian species were aligned with that of hsCAD. Alignment shading and amino acid numbers are as for Fig 4. The hsFIH preferred target sequence is indicated below the alignment using the same colouring as in Fig 3.
(TIF)

S1 Table Species name abbreviations and sequence accession numbers. List of species examined, with taxonomic name abbreviations used in various figures. Accessions of sequences (in NCBI, Uniprot, or Ensembl format), used to infer the conservation of FIH, HIF-α/CAD, PHD or Notch homologs in different species together with BLAST support data are shown, as well as accessions of sequences cloned in this work.
(XLSX)

S2 Table Primers used for cloning. All primer sequences are depicted 5’-3’. Restriction sites are underlined.
(DOCX)

S1 File Alignment of representative FIH homologs. Alignment of full length FIH sequences generated and depicted as for Fig 5, except that species names are coloured as for Fig 3 to indicate the “CAD type” found in that organism. Species name abbreviations and sequence IDs can be found in S1 Table.
(PDF)
