# Supplementary figures and images for: The conservation and functionality of the oxygen-sensing enzyme Factor Inhibiting HIF (FIH) in non-vertebrates
Source: PLoS One. 2019 Apr 29;14(4):e0216134. doi: 10.1371/journal.pone.0216134 (PMC6488082; doi:10.1371/journal.pone.0216134)

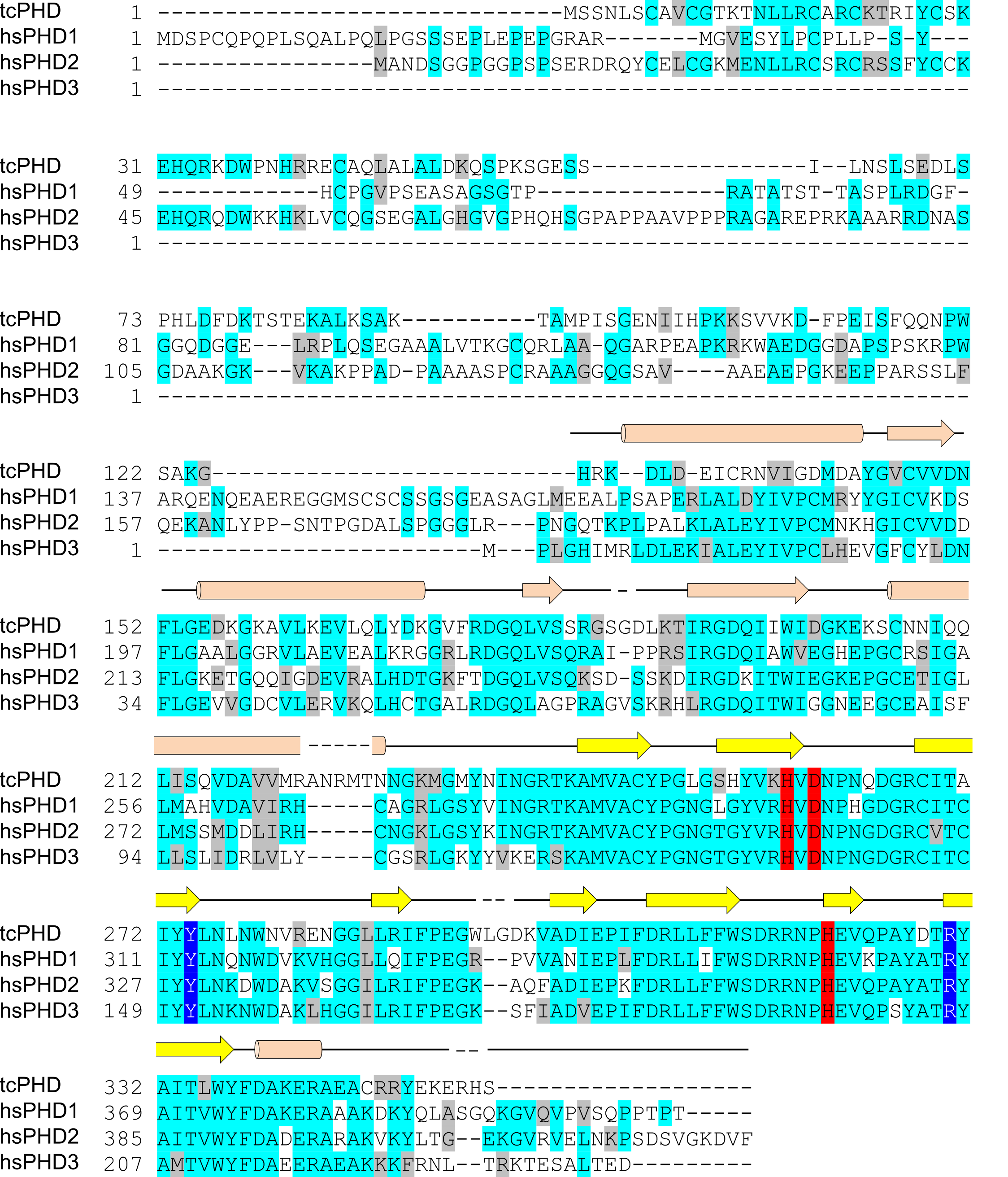

Supplement: S1 Fig — The three human HIF PHDs were aligned with T. castaneum PHD using Clustal Omega [51]. Residues strongly or partially conserved are shown in cyan and grey, respectively. Iron coordination (red) and 2-OG binding residues (dark blue) are also indicated. The structure of the catalytic domain of hsPHD2 is indicated above the alignment [53], with yellow arrows indicating the β-strands that comprise the DSBH. (TIF) [file pone.0216134.s001.tif]

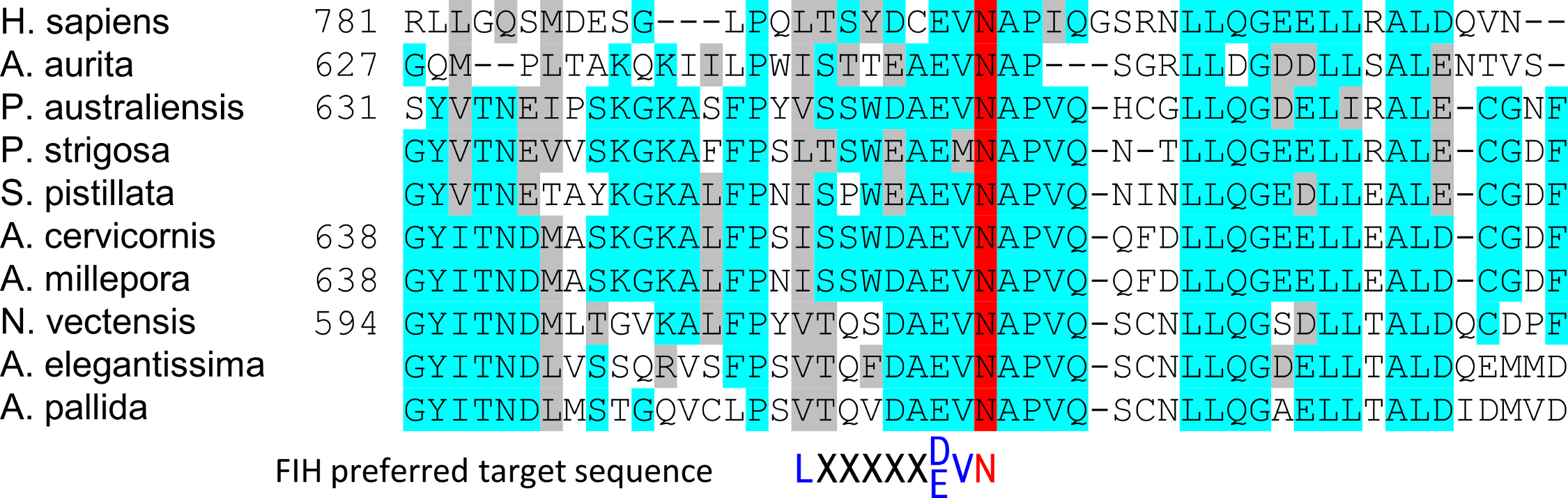

Supplement: S3 Fig — Predicted CAD sequences from a variety of cnidarian species were aligned with that of hsCAD. Alignment shading and amino acid numbers are as for Fig 4. The hsFIH preferred target sequence is indicated below the alignment using the same colouring as in Fig 3. (TIF) [file pone.0216134.s003.tif]
