# Supplementary material for: The conservation and functionality of the oxygen-sensing enzyme Factor Inhibiting HIF (FIH) in non-vertebrates
Source: PLoS One. 2019 Apr 29;14(4):e0216134. doi: 10.1371/journal.pone.0216134 (PMC6488082; doi:10.1371/journal.pone.0216134)
Supplement: S2 Table — All primer sequences are depicted 5’-3’. Restriction sites are underlined. (DOCX) [file pone.0216134.s005.docx]

**S2 Table. Primers used for cloning.**

| **Sequence** | **Forward** | **Reverse** |
| --- | --- | --- |
| tcFIH | CCACTCATGATGGATGGTGATAAGAAA | GGACTCGAGTCATTCAGTATACCTAC |
| tcHIF-α | CCTACTAGTATGGAGTGTAGTACTTTTCCACAAGACG | CCAGCGGCCGCCTAAATGGGCCCGTTGATGTCAAGC |
| tcHIF-α (790-879) | CCAAGGTACCAGTGACAGTGTCTTGA | GAACTCGAGCTAAATGGGCCCGTTG |
| tcNotch (1747-1989) | CCAAGGTACCAATGATGTTAACGCAC | GGTCTCGAGTTAATCCAAAAGTCGCA |
| tcPHD (2 – 356) | CCTAAGCTTACCATGGCTAGTTCAAATTTAAGTTGCGCGGTGTGC | CCACCTCGAGTGAATGCCGTTCTTTTTCGTATCTCCG |
| amFIH | gaaccATGGCATCGGTTGTCAGTGATA | gttctcgagctatgagagtacatctgctcca |
| am HIF-α (604-693) | ccaggtaccgctccaaccaaggctgttcc | ggtctcgagtcacaccacataaagagaagag |

All sequences are depicted 5’-3’. Restriction sites are underlined.
