# Supplementary material for: The conservation and functionality of the oxygen-sensing enzyme Factor Inhibiting HIF (FIH) in non-vertebrates
Source: PLoS One. 2019 Apr 29;14(4):e0216134. doi: 10.1371/journal.pone.0216134 (PMC6488082; doi:10.1371/journal.pone.0216134)
Supplement: S1 File — Alignment of full length FIH sequences generated and depicted as for Fig 5, except that species names are coloured as for Fig 3 to indicate the “CAD type” found in that organism. Species name abbreviations and sequence IDs can be found in S1 Table. (PDF) [file pone.0216134.s006.pdf]

Supplementary File 1

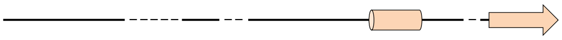

```
P. brassicae        1 --------------------------------------MLWAKRS--------DRSVPVD
E. huxleyi        306 --------------SPPAWPA--------------------------H-----YRPIAHE
B. natans          74 -----------------------------PKRYKESLETRPSDDTLDSYR--NIRPVCVV
G. theta            1 ------------------------------------------MACVP---PFPCQPVQRI
Chrysochromulina  138 --------------DPHAWPEV------------PSAAEHLPPDCFRSVHCAPMRSVPRC
C. owczarzaki       1 MDQVASHISQQIERGRLRLPGV-----------------SSPVVNCRQLGV-ATQPIPRY
O. cincta           1 -------MSETPFRDCLGGPGTP-----VPQPFLNNGTGHYDGGDTHNYNF-ITGNVPCY
S. rosetta          1 ----------------------------------MSGGGEW-----WQCPC-PLESVPRV
L. anatina          1 -------MAT--AEVPSDG-----------RKPHEKKESGYYTSQMFDYFF-DTEDIPCL
C. gigas            1 -------------------------------MAKE-----KKNFDLKEYPF-STEEIPRL
L. gigantea         1 -------MATSVERSPESG-----------EDAYS-----TIRGELKQYSF-QTEPVPRL
A. queenslandica    1 -------------------------------MAEPKGPPSSSKIEMKKYSF-PVKPIQRL
A. aurita           1 -----------------------MEDSPRSANKTQPDYCLIDGHRVRNYNI-KTTEIPRL
C. intestinalis     1 --------------------------------MAVFASGSYDPDSITKYSS-ITEQIPRR
N. vectensis        1 ------------------------------MAAVSDLYTDLDGHLVRKYSL-DVIQVPRL
A. millepora        1 ------------------------MASV--VSDKRGTYVDLEGHKVRKYDFLQLSKIPRL
S. purpuratus       1 -----------------ASSASECGTSK--ADQNTKCRIDLDPLQHRKYTF-PSEQIPRL
O. bimaculoides     1 -------------------------------------MAAEAGDPPHRYKF-PLSKVPRL
D. citri            1 -----------------------MA-----ESSQTDSDGKSSTDGFRKYNI-PLEQVPRL
T. cancriformis     1 ------------------------------MESGEISSPAKKIPGLKYYTF-IEGKVPIC
C. teleta           1 ------------------------------MATVEGGTDGWDENQLRKYTF-PTTQLQRM
A. pisum            1 -------------------------------------MAGWNESQLRKYNI-KVDKIPTL
H. sapiens          1 -------MAATAAEAVASG-----SGEP--REEAGALGPAWDESQLRSYSF-PTRPIPRL
B. floridae         1 ----------------------------------MAGAEGWSSAQLRQYSF-PLETIPRL
F. occidentalis     1 ---------------------------------MSTSRNEPDQCNLRKYDF-PLDDIPRL
C. aquilonaris      1 --------------------------------MATAIGNGW--GDQKDYGF-PTVPIPRM
I. scapularis       1 ---------------------------M--AQSECEGCTGCSSLDFQDYGI-PLEAVPRL
A. geniculata       1 --------------------------------------MADSKYHFRRYNF-PVEPIPVL
C. sculpturatus     1 --------------------------------------MASPQTELKSYDF-PLEPIPRL
S. olivacea         1 -----------------------MEESV--EHKQHVVNHNHTAHHLKQYFF-PTEHVPRL
T. castaneum        1 ---------------------------------MDGDKKPWDPSQLRKYDL-NLEEIPRL
S. maritima         1 ------------------------------------MAVSESKVAFRQYDF-PKQSIPRL
E. danica           1 -----------------------MAYAP--YLMASEEKRGWHPDQLRKYDL-NIEEIPRL
R. prolixus         1 --------------------------------VMDQERRVWDETQLRKYDI-SLKSVPKY
P. humanus          1 --------------------------------MAENNEKNWDQSQLRKYNI-QCDQIPRL
C. pallens          1 -------------------------------MKMAQNNIEWNENQLRKYDF-ILEDIPRL
Z. nevadensis       1 ----------------------------------MADKKEWDQSQLRKYDI-QLDEIPRL
T. commodus         1 -------MRRRAAPHILRGPPVESSTVR--TGTKMAQKRGYDASQLRKYDI-QLEEIPRL
```

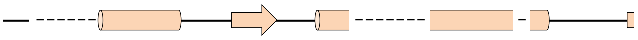

```
P. brassicae       14 DV---AADVVDAGDAAIQADRPVVVDGLAS----------RAGDWTVAK-LLDAIGDVQV
E. huxleyi        321 PFGDGGQATLDTLARYVEADQPVVLTGADIVDV---------ERWSDNAKVEALLGDKAV
B. natans         103 PWG------SPLLDQFLEAHLPIVIRGSQLIKRGGSTGESKESKWTPGH-IQSMLAAKKV
G. theta           16 SFD------DPAVIDFLRREEPVILSNVSLVRP-------LVGKWNVDY-LKEIFPMISS
Chrysochromulina  172 PFG------SKAARALLEQRKPVILTHHTLVGG-------AASKWTLDY-LCQQMHGLPC
C. owczarzaki      43 RAT------DDAARLALRSKTPFVLLDSSIAAP-------AVERWDLEF-LKEHLGMSN-
O. cincta          48 DFN------DPVALNLIKEQVPVILKGADLVSS-------A-MKWDLDY-LSTHIGGGRF
S. rosetta         21 RVC------DPKLLDFIREQRPIVITDSKLVES-------A-RHWDLEY-MEKHMGDCKF
L. anatina         40 NAST----QSSKVDQFIASERPVLMTNTDLVKT-------A-LHWDLNY-LEQHLGDGDF
C. gigas           24 NID------NPETEKRIAAGLPVVITDSGLVRS-------A-LHWDLDY-LQGNIGDGKF
L. gigantea        37 HHL------DPRVEALMRAEKPVVITDTNLVKS-------A-FHWDADY-LSSNIGSAKQ
A. queenslandica   29 CWT------EATADHLMTHEEPVVLTDTDLVQS-------A-LHWDLSY-LEQNMGPAKH
A. aurita          37 NFN------DPEAAELISSQKPVVLTGTNLIEP-------A-LHWNLEY-LRDNIGDGEN
C. intestinalis    28 SIQ------DPETYQLIKDSKPVVITDTNLVTS-------A-SKWDLEY-LNKNLGDGLF
N. vectensis       30 HYQ------DPKATEIIKNECPVVLTDSDIISS-------A-MKWDIEY-LRENIGDGDF
A. millepora       35 EYD------DPNVESIIANEEPVLLVNSNIISS-------A-LKWDLDY-LKKNLGAGSF
S. purpuratus      41 DVS------DPKVSEYVRDGKPVVITGTNLVET-------A-LKWDLDY-LEKNLGSGKF
O. bimaculoides    23 PYD------DPQAQRLISAEQPVVLTGSDLVDS-------A-LHWNLDY-LSDNMANTQF
D. citri           32 SFD------DPRVDEYIGDMKPVLITGSNLIQS-------A-MKWDLEY-ISEHMPNSNL
T. cancriformis    30 DAC------DPEALNLISKQFPVLLRNTRLIDS-------A-LHWDIEY-LERHAGSSDF
C. teleta          30 SCK------DPRLNDVIAREEPVVITDCNLASS-------A-SHWSLEY-LSSNIGNGTF
A. pisum           23 QYD------DPKVDSLLSNNKPVLIKGSKLVSQ-------V-LKWDLDY-LAEHMNSICC
H. sapiens         46 SQS------DPRAEELIENEEPVVLTDTNLVYP-------A-LKWDLEY-LQENIGNGDF
B. floridae        26 SCT------DPEADRLISEEKPVVLTDTHLIDS-------A-LKWDLGY-LRANLGGGLC
F. occidentalis    27 NCM------DPQVDELIKSKKPVVLTGSNLVGA-------A-SKWDLDY-LEKNMGNGDF
C. aquilonaris     26 SCH------DPEAQRRIFAMQPVVLTDTNLVTS-------A-LKWDLDY-LESNIGGGDY
I. scapularis      31 SHT------DPEADRLIANMMPVVLTDTGLVAP-------A-LKWDLDY-LSEHLGEGSC
A. geniculata      22 SPS------DPEAEHLIASMKPVVLKNTNLVSS-------A-LKWDLDY-LEANIGDGVF
C. sculpturatus    22 SCL------DPNADALIENMKPVVLTDTNLVSS-------A-LKWNLDY-LQKNLGDGSF
S. olivacea        35 SAS------DPLASQLIAEEKPVVLTDTNLCDT-------A-LKWDLDY-LAQHMGSERY
T. castaneum       27 HYK------DPKIDEYIKENKPVVITESNIVKP-------AVQRWSLEY-LERNLGHSGH
S. maritima        24 SIF------DPEADKLISEERPIVLTDSNLIRP-------A-LKWDLEY-LESHLGPGNY
E. danica          35 EYS------DARVDEFIAKDRPVVITGTCLVAP-------A-AKWTLEY-LAHNIGQGEF
R. prolixus        28 KVS------DPKVEELIAARKPVIIVDSDLVAP-------S-TKWDLNY-LQEHMGSTNC
P. humanus         28 RFD------DPKVLELILQNKPVVILGSDLVKS-------T-EKWDLEY-LEKNMGDSDF
C. pallens         29 DCN------DPNVDILISSNRPVVITGANLVNT-------A-NKWNLEY-LEKNMGNSDF
Z. nevadensis      26 SVK------DPKVDEFIADNKPVIITDTRLVAS-------A-ERWDLDY-LEQNMGNGDF
T. commodus        51 HAS------DPRVDELISENKPVVITGTDLVSS-------A-MLWDLEY-LERNMGNGDY
```

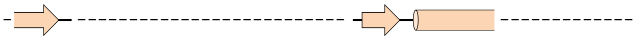

```
P. brassicae        61 RNVFISE-------------------------QSARFLYFKADRAGDTS-----------
E. huxleyi         372 -LVKRAR--------------------------GRRFRYWANDDPGARTAPGGGGGGGGG
B. natans          156 -TVYESP--------------------------SQYFRYWKKEQHTGP------------
G. theta            62 -SPFTSE--------------------------SNFFQYWDEEKNAGQ------------
Chrysochromulina   218 -TVYSSS--------------------------SRHFRYWDDEKNQAGYP----------
C. owczarzaki       88 -TVFVSS--------------------------TGKVKYFDETRLTPGQEA--S------
O. cincta           93 -AVFIRNAEADRHALVQPQSSSNPQSQSVQPEPLHIFKYFDEKKLVALQD----------
S. rosetta          66 -TVYESK--------------------------ERVFMFSDEEKNLG-------------
L. anatina          87 -TVYTSD--------------------------NDKFLYHDEKKMEA-------------
C. gigas            69 -TVYKSK--------------------------NKRFQYFDDKKVDT-------------
L. gigantea         82 -TVYSSY--------------------------KNKFLYYDDKKLPQ-------------
A. queenslandica    74 -TVYVSK--------------------------SRYFMYYDERKVND-------------
A. aurita           82 -AVYLSE--------------------------NGKFMYFDENRCKG-------------
C. intestinalis     73 -YVYTSN--------------------------DNRFKYFDNKRAEK-------------
N. vectensis        75 -AVYASE--------------------------DNKFMYYDEKRVKNW------------
A. millepora        80 -SVYSSK--------------------------TCKFMYYDEKRAKQW------------
S. purpuratus       86 -NVYVSK--------------------------NHHFMYFDDKKASN-------------
O. bimaculoides     68 -TVYQSD--------------------------NNYFKYYESRKIPQ-------------
D. citri            77 -TVIISK--------------------------DHNFKYVNEKKLPQWPS----------
T. cancriformis     75 -TVFQSQ--------------------------SSKFKYYDEQKIKD-------------
C. teleta           75 -SVYESD--------------------------SHLFKYFDEKKIPG-------------
A. pisum            68 -NVLVSK--------------------------NHKFKYYDQKKITP-------------
H. sapiens          91 -SVYSAS--------------------------THKFLYYDEKKMAN-------------
B. floridae         71 -SVYESD--------------------------NHKFMYFDEKKAKD-------------
F. occidentalis     72 -TIFVSK--------------------------GNQFKFFDEKKLAEIDSV--SGAHTKN
C. aquilonaris      71 -VVFISK--------------------------GHMFKYYDDKKVGA-------------
I. scapularis       76 -TVYQSD--------------------------TPYFKYYDETKVRDH------------
A. geniculata       67 -TVYESD--------------------------RHVFKYYDDAKYQE-------------
C. sculpturatus     67 -TVYISK--------------------------NHKFKYYDDTKIQE-------------
S. olivacea         80 -MVFLSN--------------------------NHKFKYYDEAKIKQ-------------
T. castaneum        73 -TVFVSR--------------------------NHKFKYYDEKKIYNRVSN--T------
S. maritima         69 -TVFQSK--------------------------TCKFKYYDDTKVQDE------------
E. danica           80 -TVYFSK--------------------------NHVFKYFDEKKVKD-------------
R. prolixus         73 -TVISSK--------------------------NHKFKYYDEKAISPA------------
P. humanus          73 -TVFQSR--------------------------NHLFKFFDDKKVNQLLS----------
C. pallens          74 -TVFVSR--------------------------NHIFKYYDEKKITRPLAN--G-----E
Z. nevadensis       71 -SVFLSR--------------------------NHKFKYFDDKKILPTSG----------
T. commodus         96 -TVFLSR--------------------------NHKFKYFDDKKISQVAG----------
```

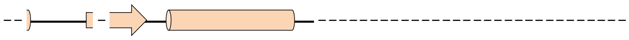

```
P. brassicae        85 -------PHLQRAPPMRFREFVERSQAACS------------------------------
E. huxleyi         405 GGAFGFEPP-TEEASLSFGEFLAEAERLA-------------------------------
B. natans          176 ---YVFKAP-TSEKSITISEFLQRVQETTKEGPAID------------------------
G. theta            82 ---YEYTPP-TSKIQMNFHDFVETIRRP--------------------------------
Chrysochromulina   241 ----FAADDHTAKLSMDIDEFRRRLAGLASTGSAA-------------------------
C. owczarzaki      111 -------HHDVRKLTMRFAEFLECMAVAARQTERFAPRPQSSCNEPTTSTQRDSCCQPTA
O. cincta          142 --RSTFQST-MVRVEMTFQEFLKRFHGD--------------------------------
S. rosetta          86 --NYKFTPT-ATKRSMTFQEFASEFREA--------------------------------
L. anatina         107 --VTNFKPP-TKRREMKFPEFVSKIKSW--------------------------------
C. gigas            89 --FKDFEKP-MEHLDLTFPEFVKKLKTA--------------------------------
L. gigantea        102 --FPDLQLA-TKHEEMTFKEFTEKVKNH--------------------------------
A. queenslandica    94 --FPSFKEP-MKKREWTFSKFVDEYKSH--------------------------------
A. aurita          102 -KYVNFQPP-TQKLSLTFYEFYELLRSW--------------------------------
C. intestinalis     93 --CKSFVTP-SERHHVTFLQFLQMLKRC--------------------------------
N. vectensis        96 ---PHFRPP-TVRIDMKFEQFYKQVNKF--------------------------------
A. millepora       101 ---PYFIPP-TQRIHMKFEEFCSRVRNL--------------------------------
S. purpuratus      106 --QKEFSPK-TRLMEMTIPEFVQLMEEK--------------------------------
O. bimaculoides     88 --QKDFVPP-TIQMQMSFSEFVEKLKEA--------------------------------
D. citri           100 ---STLEPITTRWSSMTAQEFSTRIKSW--------------------------------
T. cancriformis     95 --CPGFVPS-ITKLEIPFSNFAARLRQW--------------------------------
C. teleta           95 --HKDFRPE-MRRKEMKFNDFVALMQNP--------------------------------
A. pisum            88 --NMTFKPI-SRPSPMIFSEFAKKIKDW--------------------------------
H. sapiens         111 --FQNFKPR-SNREEMKFHEFVEKLQDI--------------------------------
B. floridae         91 --RKDYTPC-TKRMEMPFEEFVKQLESS--------------------------------
F. occidentalis    103 RPQKEYIRP-AKSCPIKIQDFAKRVREW--------------------------------
C. aquilonaris      91 --RSKFIPP-TQRIDLKFPVFSQRLKEW--------------------------------
I. scapularis       97 -RLTDFRAP-TLRHEWKIGTFAQRLRSA--------------------------------
A. geniculata       87 -HNLDFHPP-AERYDMKFPEFSKKLRNS--------------------------------
C. sculpturatus     87 -HCLDFTAP-TERFDMKFVDFVKRLRKL--------------------------------
S. olivacea        100 -YKTNFVPP-TRRVDLTFPEFVKKLREW--------------------------------
T. castaneum        98 -KGVEFTPP-TRKVEMRIEDFMKRVKEW--------------------------------
S. maritima         90 RIFADFVPP-TRRIDMKFPEFAVKLRNW--------------------------------
E. danica          100 -YTGKFNPP-THRLDMKFPEFVRYMKQW--------------------------------
R. prolixus         94 -IKAKFTPP-TKKVSMKMAEFAKRLREW--------------------------------
P. humanus          96 NYKMEFTPP-TKRIDIKLSEFCQRLREW--------------------------------
C. pallens         100 IYKVDFVPP-TTRRDMKISEFITKLKQW--------------------------------
Z. nevadensis       94 DSRLDFTPP-TRRVDMKLPEFTRKLRQW--------------------------------
T. commodus        119 ESKAEFTPP-TRRVDMKLPEFSQRLKQW--------------------------------
```

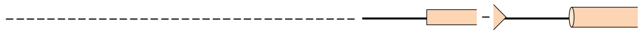

```
P. brassicae     108 -------------------------------------TGRHYYLYGEPLP----------
E. huxleyi       433 ----------------------------------AGDSAERLYLQ-ETMSGH--PELAEE
B. natans        209 ------------------------------EDGNKKGRSGLLYLQ-ESLSGH--SEFSSE
G. theta         107 ----------------------------------QSHAGRRHYLQ-QMLVTGMGERILDD
Chrysochromulina 272 -----PAE--GVDSKDS---AAGD--GGDAAGGDASSGSQRLYLQ-TALVDGVGAEMMAD
C. owczarzaki    166 AAAVAPADAAAFDSNSQQPPAASDDSAARIASQFGIHSGDVVYLQ-QGLSNSVGLKIVES
O. cincta        167 ------------------------------------RSQERVYLQ-QPLNNTVGPQIASD
S. rosetta       111 ----------------------------------MKSKDKFLYLQ-QGLNDTVGPTIVKD
L. anatina       132 -----------------------------------KKGSEKNYLQ-QTLNDAVGKRIVID
C. gigas         114 -----------------------------------KPGKDKVYLQ-QALNEGVGKRIVAD
L. gigantea      127 -----------------------------------KKGDKIYYLQ-QALNDSVGKSLVLD
A. queenslandica 119 ----------------------------------EGRTDEYLYYQ-DALTDTVGSGIKHD
A. aurita        128 -----------------------------------KEGDKKIYFQ-QMLTNRVKDKVVTD
C. intestinalis  118 -----------------------------------ARTKEKVYLQ-QTLNDSVGTNIVTD
N. vectensis     120 ------------------------------DPTMAKAGNLRFYLQ-QMLNDQVGRNIVAD
A. millepora     125 -----------------------------------KASDSRVYLQ-QMLDDSVGKNIVKD
S. purpuratus    131 -----------------------------------KGGTDKVYLQ-QALNDSVGPSIVED
O. bimaculoides  113 -----------------------------------QCSKKQLYLQ-QPLTEGVGDKIVQD
D. citri         125 -----------------------------------KEGDERLYLQ-QVLDSSVGPQLVQD
T. cancriformis  120 -----------------------------------KIGHLRIYLQ-QALSSTVGEGIVLD
C. teleta        120 -------------------------------------EQRRFYLQ-QPLNDTVGPQVVKD
A. pisum         113 -----------------------------------KKGDSRLYMQ-QVLNNSISQRIVSD
H. sapiens       136 ---------------------------------QQRGGEERLYLQ-QTLNDTVGRKIVMD
B. floridae      116 ---------------------------------AETE-GKRVYLQ-QTLNDTVGKNIVVD
F. occidentalis  130 -----------------------------------KDGDERLYLQ-QALSSTSGAGVVQD
C. aquilonaris   116 -----------------------------------KPGKERLYLQ-QALNNTVGQGIVQD
I. scapularis    123 -----------------------------------G-HNKYYYLQ-QMLNETVGQSIMLD
A. geniculata    113 -----------------------------------ELGGRRIYLQ-QPLNDTVGKNIVVD
C. sculpturatus  113 -----------------------------------QANDRRLYLQ-QPLNDTVGKNIVKD
S. olivacea      126 -----------------------------------KPGDERVYLQ-QGLNNTVGQAIVMD
T. castaneum     124 -----------------------------------KKGDERIYLQ-QSLTTTVGNNIVED
S. maritima      117 -----------------------------------KSGDDRIYLQ-QTLNSTVGQAIVED
E. danica        126 -----------------------------------KAGNDRPYLQ-QALNSTAGPSIVED
R. prolixus      120 -----------------------------------KKGNDKLYLQ-QTLNDTVGPSIVRD
P. humanus       123 -----------------------------------KKGDDRLYLQ-QLLNNTVGPAIVDD
C. pallens       127 -----------------------------------KRGDDRLYLQ-QALNTTVGSSIVQD
Z. nevadensis    121 -----------------------------------RRGEERMYLQ-QALNNTVGPAIVQD
T. commodus      146 -----------------------------------KRGEERMYLQ-QALNNTVGPSIVQD
```

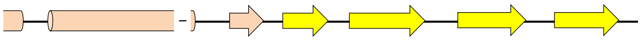

```
P. brassicae     121 --SALWRSLPDMERVLGSTAQLTSSLLWMALDATCSPLHYDMCHGLLLQMAGQKRFIMFP
E. huxleyi       456 FRSWNWPFLLQNSNRH-RWGLPDSNELFIGMQGAETDAHYDERHNLFFQVRGRKQLLLFP
B. natans        236 FASWNWNWLLRIVQKY-GWGTPDRNILLIGMQGATTPAHYDEQENMFAQMRGRKEVYLWP
G. theta         132 FKHVDWGTVYGWKAQL-GFGDLTTNLLLVGTTGHVTPAHYDEQHNFFCQLQGRKQVMLFS
Chrysochromulina 319 FRAFDWEGVLATQQRL-GWGSLSSNLLLVGQKGNTTPCHYDEQQNLFAQLHGAKRVILFS
C. owczarzaki    225 FLQFNWKLLAELQRDA-NWSDLTTNLLLVSMAGAVTPAHYDEQENLFAQVRGAKRCVLFA
O. cincta        190 FIQFNWSWAAEHKREF-NWGPLTSNLLLIGQKGNITPCHFDEQQNLFCPLRGRKRILLFN
S. rosetta       136 FVGFNWTWLTDVVKAL-NWGHLTTNMLFVSMPHLVTPCHYDEQENFFAQVRGTKRVILFH
L. anatina       156 FLGFNWDWINKQQNGN-NWGPLTANLLLIGQEGNYTPVHYDEQHNLFAQVQGCKRIFLFP
C. gigas         138 FLGFNWSWVTEQQKKN-KFGALTSNLLLIGMEGNVTPAHYDEQENFFAQIRGYKRFILFH
L. gigantea      151 FIKFNWSWATEQQKKY-KWGALTSNLLLVGMDGVITPAHYDEQQNFFAQIGGFKRFLLFD
A. queenslandica 144 FVHFNWHWVSERKKRY-NWGQLTSNLLLISQPGNITPCHYDEQHNFFCQVRGLKRCLLFA
A. aurita        152 FLSFNWGWLGVQQQRY-NWGPLTSNLLLVSQPGNITPLHYDEQENFFAQASGFKRCMLFP
C. intestinalis  142 FLQFNWDWVNKCQAAY-GWGSLSSNLLLIGLEGNITPVHYDEQENFFAQISGHKRCLLFS
N. vectensis     149 FLGFNWAWLNTMQKEM-DWGPLTSNLLLIGLPGNITPVHYDEQQNLFCQVTGCKRVLLFH
A. millepora     149 FLEFKWQWLTEMQRKM-NWGSLTSNLLLIGLPGNITPVHYDEQQNFFGQLTGFKRVILFH
S. purpuratus    155 FVRFNWNWVTDYQKKN-SWGPLTSNLLLVSMEGNVTPAHYDEQENFFAQVKGYKRFIMFP
O. bimaculoides  137 FVSFNWRWISMQQKEN-NWGPLSSNLLLVGMAGNITPVHYDEQQNFFAQLRGVKRCLLFA
D. citri         149 FLHFNWDWAKNKQKIH-GWGPLTSNLLLLNIAGNVTPCHYDEQQNLFVGIRGFKRCILFP
T. cancriformis  144 YVKFNWPWIVNIQKRN-GWGSLTSNLLLISQGGNITPSHYDEQHNLFAQIRGYKRFIFFP
C. teleta        142 FLQFNWDFAKEQQKRN-GWGPLTSNLLLIGMAGNVTPCHYDEQENLFAQVRGYKRVILFP
A. pisum         137 FVMFNWDWIMSKQKVC-KWGSLTSNLLLIAQEGNVTPCHYDEQQNMFASIRGYKRFILFP
H. sapiens       162 FLGFNWNWINKQQGKR-GWGQLTSNLLLIGMEGNVTPAHYDEQQNFFAQIKGYKRCILFP
B. floridae      141 FLQFNWDWINKQQQKQ-NWGHLTSNLLLIGMAGNVTPVHYDEQQNFFAQVKGFKRCILFA
F. occidentalis  154 FIRFRWDWVTAKQKLH-NWGKLTYNLLLVGLEGNVTPCHYDEQENLFAQIHGHKRCILFP
C. aquilonaris   140 FLGFNWPWINGQQKKH-GWGPLSSNLLLVGLGGNVTPAHYDEQENFFAQVRGHKRCILFP
I. scapularis    146 FLRFNWDWVNRQQKHN-GWGPLTSNLLLVGTGGNVTPAHYDEQQNFFAHLRGHKRFLLFS
A. geniculata    137 FLDFNWKWICEIQKQN-NWGPLTSNLLLVSSAGNVTPAHYDEQQNIFAQIRGFKRFILFP
C. sculpturatus  137 FLDFNWNWISGQQQQH-NWGPLTSNLLLIGSEGSVTPAHYDEQQNFFAQIYGFKRFILFC
S. olivacea      150 FLQFNWQWLNMQQKNN-NWGPLTSNLLLVGMEGNVTPVHYDEQQNFFSQLVGYKRCILFA
T. castaneum     148 FVKFDWDYVNGKQTKH-NWGPLTSNLLFIAMEGNQTPCHYDEQENFFAQVQGYKRCILFP
S. maritima      141 FVRFNWNWANGRQKKN-NWGSLTSNLLLIATEGNITPCHYDEQQNFFAQVRGSKRVFLFP
E. danica        150 FRNFNWDWVNNLQKKH-TWGPLTSNLMLIAMEGNVTPCHYDEQQNIFAQVYGYKRCILFH
R. prolixus      144 FLGFKWDWINGIQKKM-NWGPLTSNLLLIGMEGNVTPCHYDEQENLFAQVRGYKRIILFP
P. humanus       147 FLHFKWDWLSNIQKLS-NWGPLTSNLLLISMEGNVTPCHYDEQQNLFAQITGYKRCILFP
C. pallens       151 FRGFNWSWVNVKQKAN-SWGPLTSNLLLISMEGNVTPCHYDEQQNFFAEVRGHKRCILFP
Z. nevadensis    145 FLNFRWEWINSKQKMH-SWGPLTSNLLLIAMEGNVTPCHYDEQQNFFAEVRGFKRCILFP
T. commodus      170 FLNFRWEWINAKQKAN-NWGPLTSNLLLISMEGNVTPCHYDEQQNFFAEVRGYKRCILFP
```

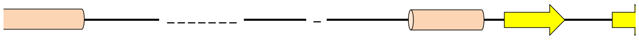

```
P. brassicae       179 PDGIPDAYMHPVGHARMGLVSDDDRQSQINDICHPDLAAFPAFAGLRGTQGVVEPGQALV
E. huxleyi         515 FVEYEKLYPFPVTHP-------CDRQSMV-DVARPDLEAFPAFATASGQYTTLQPADLVF
B. natans          295 PGDWQGMYAFPCNHA-------CDRQAMV-NPDNPDLHRFPLYRHARPHYAVLQPGDLLY
G. theta           191 PAEWGKMYPFPLHHA-------CDRQSMM-P--SHDRMLFPNFDRASGYEAIVHPGEMLY
Chrysochromulina   378 PADFGCLYPFPMHHP-------CDRQSQV-DLYAPDLSRFPRFRAARPLEAILQPGEVLY
C. owczarzaki      284 PDRFPCLYPYPVHHP-------CDRQSQV-DFDNPDLARFPRFSELHGWECILEPGEVLY
O. cincta          249 PENFPYLYPYPVHHP-------NDRQSQV-NLDNPDYGRFPKFAMARGVQATVGPGEILY
S. rosetta         195 PDNFRCLYPYRYGHP-------CDRQSQV-DFDNPDYERFPKFKDARGLEAILRPGDVLY
L. anatina         215 PSQFECLYPYPVYHP-------CDRQCQV-DLRAPDFDRFPKLKRLKGYEVIVKPGDVLY
C. gigas           197 PDQFKCMYPYPTYHP-------CDRQSQV-DFDNPDYKRFPKFKDVKGYETVVGPGDVLF
L. gigantea        210 PSMFECLYPYPVYHP-------HDRQSQV-DFDNPDHERFPNFRNLYGQEAIVGPGDVLY
A. queenslandica   203 PDQYDKLYPYPVAHP-------CDRQSQV-DFDSPDFERFPKFKEIEGYECILSPGDVLY
A. aurita          211 PSQFKCFYPFPYHHP-------CDRQSQV-DFDNPDHGSFPLFKKAVGREVIVGPGDVLY
C. intestinalis    201 PDQFDCLYPHPVAHP-------CDRQSQV-NIANPDYKKFPRFRNAHATEAIVGPGDVLY
N. vectensis       208 PDKFKCLYPFPVHHP-------CDRQSQV-DFDCPDYIRFPLFKEICGMEAMVKPGDVLY
A. millepora       208 PDQFKCLYPFPLYHP-------CDRQSQV-DFDNPDFKRFPKLKELKGYEVIVGPGDVLY
S. purpuratus      214 PSQFDRLYPFPVHHP-------NDRQSQV-NFDDPDFERFPKFRNAKAVEAVVGPGDVIY
O. bimaculoides    196 PDQFKSLYPFPVYHP-------HDRQSQV-NLENPNLEKFPKVLELKGFEATLHPGDVLY
D. citri           208 PEQFDCLYPPPVSHP-------HDRQSQV-DFENPDLQRFPRFREAKGMEAVVGPGDILY
T. cancriformis    203 PEQFENLYPHPFPHP-------HDRQSQV-DFDEPDYIRFPNFHKARGLEVVVGPGDVLY
C. teleta          201 PEQFSCLYPYPVHHP-------HDRQCQV-DFENPDYERFPLFKDVAGQEAVLGPGDVLF
A. pisum           196 PSEFECLYPHPVHHP-------YDRQSQV-DFDNPDYIKFPKFKEACGYEVIVGPEDVLY
H. sapiens         221 PDQFECLYPYPVHHP-------CDRQSQV-DFDNPDYERFPNFQNVVGYETVVGPGDVLY
B. floridae        200 PEQIECLYPYPVHHP-------CDRQSQV-DFDNPDYNRFPKFRYASGLEAVVGPGDVLY
F. occidentalis    213 PEQFGCLYPHPVWHP-------HDRQSQV-DLDNPDYDRFPKMRDLHGVQAVVGPGDVLY
C. aquilonaris     199 PEQFDCLYPHPVYHP-------HDRQSQV-DFENPDLSRYPKFSEASGMETILSPGEVLY
I. scapularis      205 PDQYGCLYPHPVWHP-------HDRQSQV-DFSDPDLSRFPEFAHLRGWETVLGPGDVLY
A. geniculata      196 PEQYECFYPYPVFHP-------HDRQSQV-DFENPDYIKFPKFRDAHGYEAILGPGDVLY
C. sculpturatus    196 PEMYECLYPHPVWHP-------HDRQSQV-DFDNPDFEKFPRFHEIRGQDAIIGPGDVLY
S. olivacea        209 PEHYERLYPYPVYHP-------HDRQSQV-DFDDPDMEKFPGLKHLQGMEAVVGPGDVLY
T. castaneum       207 PSQFECLYPYPVHHP-------HDRQSMV-DFERPDYNKFPKFKNVKGWEAVVGPGDVLY
S. maritima        200 PEQFECLYPYPVYHP-------HDRQSQV-DFDNPDYERFPKFREIEAMEAVVEPGDVLY
E. danica          209 PDQFDCLYPHPIFHP-------HDRQTQV-DFENPNYERYPKFPKAKAMQAVVGPGDLLF
R. prolixus        203 PEQFDCLYPHPVFHP-------HDRQSQI-DFDEPDFVKFPKFSEAKGEEAIVGPGEVLY
P. humanus         206 PEQFECLYPHPVYHP-------HDRQSQV-DFENPDLKKFPKFSQVKGQETVLGPGDVLY
C. pallens         210 PEQFECLYPYPVYHP-------HDRQSQV-DFEKPDYNRFPKLREARGMQAVIGPGDVLY
Z. nevadensis      204 PEQFECFYPHPVYHP-------HDRQSQV-DFERPDYTRFPKFREARGQEAIVGPGDVLY
T. commodus        229 PEQFECFYPHPVYHP-------HDRQSQV-DFDRPDYTRFPKFREARGQEAVVGPGDVLY
```

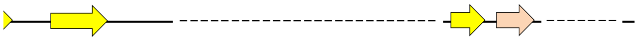

```
P. brassicae       239 IPYGWMHQIEAGPGL---------------------------SISVSTSVHA---ARTCG
E. huxleyi         567 LPYGWFHWLRNDDA---------------------------LSISLSFWSLS-------T
B. natans          347 LPVGWWHHLRNLDP---------------------------LAISITFWSKQ------QK
G. theta           241 IPSYWWHHVINLE----------------------------ETVSLTFWFK-------CP
Chrysochromulina   430 LPQYWWHHIENLDE---------------------------ACVSLNFWFK-------DC
C. owczarzaki      336 IPAYWWHHVESLT----------------------------DSVSVNFWYL-------VG
O. cincta          301 IPIHWWHHVINLD----------------------------DTIAINFWHK-------GG
S. rosetta         247 IPRCWWHLVRSLDE---------------------------LSVSVNFWYMS------PP
L. anatina         267 IPMYWFHHVESIIGSG-------------------------ITTSITFWYK-------SG
C. gigas           249 LPMYWWHQVESLPDHG-------------------------HTISVTFWYK-------AG
L. gigantea        262 LPIYWWHQVESVPNHG-------------------------MTVSVNFWYK-------SG
A. queenslandica   255 IPMYWWHTIETSPGE--------------------------LSISITFWYR-------GG
A. aurita          263 IPVYWWHYIESVMDEG-------------------------PTISINFWYKA------AA
C. intestinalis    253 IPMYWWHQIESLSSSDAMDVSHPHSLQDSASIDDVMEGIPPVTISVNFWYR-------GT
N. vectensis       260 IPMYWWHYVESTLNGG-------------------------ITTSVNFWYK-------AG
A. millepora       260 IPMYWWHHVESTMNSG-------------------------ITTSVNFWFK-------AG
S. purpuratus      266 IPMYWWHHVESLLHGG-------------------------VTVSVTFWFK-------AG
O. bimaculoides    248 IPIYWWHQIESLPQKG-------------------------VTISINFWYK-------NS
D. citri           260 IPLYWWHHIESKQKED-------------------------YTVSLNFWYK-------TK
T. cancriformis    255 IPAYWWHYVESLPGEE-------------------------CNISVTFWYK-------AG
C. teleta          253 LPMYWFHHFESLLDGG-------------------------LTTSVTFWYK-------AP
A. pisum           248 IPMYWFHHVESLMHGG-------------------------CTVSVNFWFK-------AG
H. sapiens         273 IPMYWWHHIESLLNGG-------------------------ITITVNFWYK-------GA
B. floridae        252 IPMYWWHHIESLLEGG-------------------------YTTSVNFWYK-------AG
F. occidentalis    265 IPIYWWHHIESVKPEK-------------------------YTVSLNFWYK-------AG
C. aquilonaris     251 IPVYWWHHIESLQQNQ-------------------------YTVSINFWYKTFNFAKQSR
I. scapularis      257 LPMYWWHQVESAPGKD-------------------------YTVSVNFWYK-------AA
A. geniculata      248 LPMYWWHHVESALDGG-------------------------YTISVNFWYK-------SG
C. sculpturatus    248 IPMYWWHHVESLLRTK-------------------------YTISVNFWYK-------SG
S. olivacea        261 IPMYWWHQIESLPHLG-------------------------NTVSVNFWYK-------GG
T. castaneum       259 IPIYWWHHIESLLRGG-------------------------PTVTVNFWYK-------GG
S. maritima        252 IPMYWWHHVESLQTGG-------------------------YTVSVNFWYR-------AG
E. danica          261 IPIYWWHHIESLMNGG-------------------------NTLSINFWYK-------AA
R. prolixus        255 IPVYWWHHVESLLRGG-------------------------YTVSVNFWYK-------VG
P. humanus         258 IPIYWWHHIESLMRGG-------------------------YTFSINFWYK-------AG
C. pallens         262 IPIYWWHHVESLMRGG-------------------------YTISVNFWYK-------AG
Z. nevadensis      256 IPIYWWHHIESIMRGG-------------------------YTISVNFWYK-------AG
T. commodus        281 IPIYWWHHIESIMRGG-------------------------YTISINFWYK-------AG
```

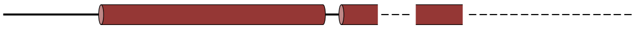

```
P. brassicae       269 LPP-------AA--CAGIAGNLL---HDAPSAVRDI-----YLR----------------
E. huxleyi         593 K-KERVPDVFSAHDLTLVRRNLEKHMAARFGAALFPQRMRRLLRLI-DA-GPGGETSDGV
B. natans          374 ASLDTLKLPLDQRHLLTVRRNLEEMVFNIFGSESLQN-LAKQLSAKH-SSKPSTKLSEG-
G. theta           266 PVG-DLRLPISQVQRVALRRNIENMFGSMLGNRNEI---SAFLQ----------------
Chrysochromulina   456 AKPEKVVLPLSASQHLAMRRNIEKLVAQKLGATKAQL-VLPLLASHTDAEQPAESAMPE-
C. owczarzaki       361 PP--EIAHPLTYQQKISVMRNIEKMLGEALGDPTQL---SSLLS----------------
O. cincta          326 GSA-PVEYPLQARQKIAMVRNIEKMLLEALQDQNEL---KELLH----------------
S. rosetta         274 AIAEDIRFPLTASQKVAMMRNIEQMLLTALGSAGEV---SDFLR----------------
L. anatina         295 PTG-KIEYPLKPQQKMAMMRNIEKMITEALNSSEEV---PPFMQ----------------
C. gigas           277 PIG-NVVYPLSPQQKVSMMRNLEKMIHQALNNTDEI---PEFMQ----------------
L. gigantea        290 TEE-KIVYPLKPQQKVAMMRNIEKMVTSAINNHEEV---PHLLK----------------
A. queenslandica   282 PVPSKITYPLSSQQKVAITRNIEKMLHEALQDHNEV---GPLLH----------------
A. aurita          292 NTSKEIPYPLRMDQKVAIMRNIEKLLGDALGDPDEV---NSLLN----------------
C. intestinalis    306 PLPDVIQYPLSPQQRVSVMRNVEKMLAAALGDAEKV---GELLR----------------
N. vectensis       288 QTPSEISFPLSSQQKIAIMRNIERMLGDALGSHTEV---GPLLN----------------
A. millepora       288 PTPSQITHPLTAQQKVAVMRNIERMLGEALGDPHEV---GPLLN----------------
S. purpuratus      294 AGPKQVEYPLTPQQKVSITRNIEKMLFEVLKDPKEV---ASLFH----------------
O. bimaculoides    276 CQQ-TVTYPLAASQKIAIMRNIEKMVTDALGDINEV---APFMR----------------
D. citri           289 PTG-DIEYPLKGHQKVAILRNIEKMVAEALQNQEEV---SHLMR----------------
T. cancriformis    283 PTG-VVKYPLKGYQKVAIMRNIEKILVEALQDPQEV---GPLLR----------------
C. teleta          281 PVG-KVEYPLKPQQKVAMMRNIEKMITEALNDQHEV---APFMR----------------
A. pisum           276 SVE-KIEYPLLDHQKMVIMRNVEKMLAEALHDPNEV---GNMLN----------------
H. sapiens         301 PTPKRIEYPLKAHQKVAIMRNIEKMLGEALGNPQEV---GPLLN----------------
B. floridae        280 PTPRNIVYPLKPVQKMAIMRNLEKMLAEALGDYREV---GPLIN----------------
F. occidentalis    293 PVG-QVEYPIKGYQKVAIMRNVEKMLAEAMQDPEEV---GALLR----------------
C. aquilonaris     286 PTG-DIVYPLQGYQKVAIMRNVEKMLLEALRDPEEV---GPLMR----------------
I. scapularis      285 PVD-KVIYPRMGHQKMAIMRNIEKVLVEALRDVNEV---GPLLR----------------
A. geniculata      276 PTG-KVKYPLQASQKMAIMRNVEKMLVEALKDVNEV---GPLLR----------------
C. sculpturatus    276 PTG-KIKYPLNGHQKMAIMRNIEKMLVEALKDVNEV---GPLLR----------------
S. olivacea        289 PTE-KIEYPLKPRQKLAIMRNVEKMLLEALREPAEV---GPLLR----------------
T. castaneum       287 PS--TLEYPLKDHQKVSITRNVEKMLLEVLQDPKEV---GPLLR----------------
S. maritima        280 PTG-KIEYPLKAQQKVAIIRNVEKMVSGALEKPEEV---GPLLR----------------
E. danica          289 PPP-PISYPLLGHQKIAIMRNVEKMLAEALQDQKEV---GPLLR----------------
R. prolixus        283 PTG-HITYPLKGYQKVAIMRNIEKMLVEALKDTEEV---GPLLR----------------
P. humanus         286 PTG-PITYPLKSRQKVAIMRNVEKMVINALADPREV---GPLFR----------------
C. pallens         290 PTG-EIVYPLKGHQKVAIMRNVEKMLLEALQDPREV---GPLLR----------------
Z. nevadensis      284 PTG-QIVYPLKDHQKVAIMRNVEKMLVEALQDPHEV---GPLLR----------------
T. commodus        309 PTG-QIVYPLQGHQKVAIMRNVEKMLVEALQDPQEL---GPLLR----------------
```

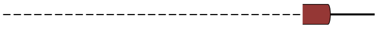

```
P. brassicae       296 ---------------------------------RMQW-------------------
E. huxleyi         650 VGEVLAEARTLLGAVQVADPEKQDEFLRSMLRVRFEGEWQAHV-------------
B. natans          431 NSKVKEFMVNCLSNF--MGRTQAEPLILDMLEGRYCNDIEKWIQPNA---------
G. theta           306 ----------------------------ELASNRFNLPVRM---------------
Chrysochromulina   514 VGALREEVTRLLTHV--MPAADVPAWLCELVEGRFPRLAHQETGQPI---------
C. owczarzaki       400 ----------------------------DLVLGRYTH-------------------
O. cincta          366 ----------------------------WIVDGRY---------------------
S. rosetta         315 ----------------------------MLVSGRYDTDANHTFRQLEM--------
L. anatina         335 ----------------------------NMVLGRYT--------------------
C. gigas           317 ----------------------------NMVLGRYTDPPA----------------
L. gigantea        330 ----------------------------TMVLGRYT--------------------
A. queenslandica   323 ----------------------------MICDGRY---------------------
A. aurita          333 ----------------------------MIVNGRYPADRKYFEEFDE---------
C. intestinalis    347 ----------------------------TMVEGRYTQ-------------------
N. vectensis       329 ----------------------------LMVNGRYNLPETESKKGETHSSHS----
A. millepora       329 ----------------------------CMVNGRYTENNDEKDYGADVLS------
S. purpuratus      335 ----------------------------LIVDGRYDLPADPMPPSS----------
O. bimaculoides    316 ----------------------------TMVLGRYT--------------------
D. citri           329 ----------------------------ALVLGRYTE-------------------
T. cancriformis    323 ----------------------------AMVLGRYTQTDAQIGA------------
C. teleta          321 ----------------------------NMVLGRYTSDDL----------------
A. pisum           316 ----------------------------TIVLGRYTSDTN----------------
H. sapiens         342 ----------------------------TMIKGRYN--------------------
B. floridae        321 ----------------------------TMIQGRYTLMDMTEVNGNKVLYTD----
F. occidentalis    333 ----------------------------TMVQGRF---------------------
C. aquilonaris     326 ----------------------------TMVLGRYTEGDQSV--------------
I. scapularis      325 ----------------------------AMVLGRYT--------------------
A. geniculata      316 ----------------------------SMVIGRYT--------------------
C. sculpturatus    316 ----------------------------AMVLGRYT--------------------
S. olivacea        329 ----------------------------SLVLGRYTGEEADRQEGALLSAATPHTN
T. castaneum       326 ----------------------------SMVLGRYTE-------------------
S. maritima        320 ----------------------------AMVLGRYT--------------------
E. danica          329 ----------------------------ALVLGRYT--------------------
R. prolixus        323 ----------------------------AIVLGRYTE-------------------
P. humanus         326 ----------------------------ALVLGRYAEASSKIDDNN----------
C. pallens         330 ----------------------------TLVLGRYTE-------------------
Z. nevadensis      324 ----------------------------ALVLGRYTD-------------------
T. commodus        349 ----------------------------ALVLGRYT--------------------
```
